# Carry-over effects of *Bacillus thuringiensis* on tolerant *Aedes albopictus* mosquitoes

**DOI:** 10.1186/s13071-024-06556-3

**Published:** 2024-11-07

**Authors:** Romina Bahrami, Stefano Quaranta, Hugo D. Perdomo, Mariangela Bonizzoni, Ayda Khorramnejad

**Affiliations:** University of Pavia, Pavia, Italy

**Keywords:** Bti, *Aedes albopictus*, Fitness, Microbiota, Immunity

## Abstract

**Background:**

The biological larvicide *Bacillus thuringiensis* subsp. *israelensis* (Bti) represents a safe and effective alternative to chemical insecticides for mosquito control. Efficient control of mosquitoes implicates continuous and extensive application of Bti. This massive use of Bti imposes strong selective pressure, but the complex mode of action of the numerous synergistic Bti endotoxins lower the risk of the emergence of resistance. Although resistance to Bti has not been identified at the population level in nature, some larvae can survive Bti exposure, suggesting tolerance mechanisms. Here we investigated whether Bti-tolerant *Aedes albopictus* larvae experience any fitness costs. We also studied how this tolerance affects different aspects of the phenotype of the emerging adults that could be relevant for arboviral transmission.

**Methods:**

We exposed *Ae. albopictus* larvae to lethal concentration of Bti and studied the fitness and gut microbiota of tolerant larvae and their adult counterparts. We further compared the transcript abundance of nine key immunity genes in the gut of Bti-tolerant larvae and their emerging adults versus those not exposed to Bti.

**Results:**

Our results showed that Bti exposure has multifaceted impacts on *Ae. albopictus* mosquitoes during both larval and adult stages. The carry-over effect of Bti exposure on tolerant larvae manifested in reduced adult emergence rate, shorter lifespan, and decreased fecundity. Bti also alters the gut microbiota of both larvae and adults. We observed higher microbial diversity in Bti-tolerant larvae and changes in the richness of core microbiota. Bti infection and the altered microbiota triggered immune responses in the larval and adult guts.

**Conclusions:**

The observed reduction in mosquito fitness and changes in the composition of the microbiota of adults emerging from tolerant larvae could negatively influence mosquito vectorial capacity. Understanding these impacts is crucial for evaluating the broader implications of Bti-based insecticides in mosquito control programs.

**Graphical Abstract:**

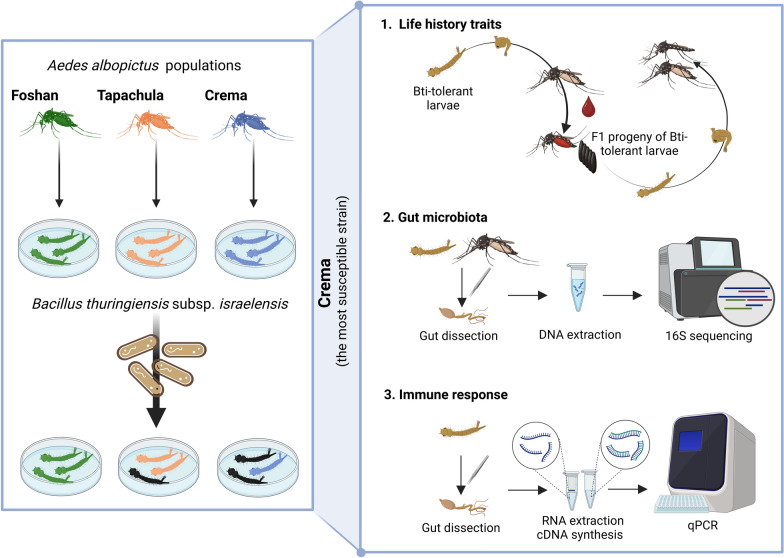

**Supplementary Information:**

The online version contains supplementary material available at 10.1186/s13071-024-06556-3.

## Background

Vector-borne diseases constitute a significant global health burden resulting in more than 700,000 deaths annually [1]. Dengue (DEN), chikungunya (CHIK), and Zika (ZIK) viruses are particularly significant pathogens that have experienced notable increases both in prevalence and geographic distributions over the past 50 years [2]. Transmission to humans of these arboviruses is primarily mediated by *Aedes aegypti* and *Aedes albopictus* mosquitoes [3]​. Given that vaccines and therapeutic drugs are unavailable or limited to few arboviruses, vector control remains the primary approach for mitigating disease transmission [4]. The use of larvicides to suppress vector populations is an important strategy, which can be carried out using environmentally safe products derived from *Bacillus thuringiensis* subsp. *israelensis* (Bti) [5, 6]. The larvicidal activity of Bti is mainly attributed to parasporal crystals produced during the stationary phase of the bacterial growth. Parasporal crystals contain four major protoxins, Cry11Aa, Cry4Aa, Cry4Ba, and Cyt1Aa, which are highly toxic to dipteran larvae [7].

Bti crystals act by ingestion. Crystals are solubilized in the alkaline environment of the midgut and processed into protoxins by larval midgut proteases. Activated toxins interact with midgut receptors, oligomerize, and insert into the membrane of midgut epithelial cells, leading to pore formation, cell osmotic lysis, and insect death [8–10]. Moreover, disruption of the midgut epithelium allows the gut microbiota to invade the hemocoel, which is a favorable environment for germination, resulting in septicemia and insect death [11].

The gut microbiota is a dynamic community [12] that can interact with *B. thuringiensis* (Bt) during the infection process [13]. The gut microbiota can affect Bt toxicity either by inhibiting its growth and degrading its toxins or by enhancing Bt pathogenicity [14–16]. In return, Bt infection can influence gut bacterial growth, resulting in an alteration of the composition of the gut microbiota [17, 18]. At the same time, modification of the gut microbiota and/or Bti itself can elicit host immunity, which may impact progression of Bt infection and influence host susceptibility to Bti [15, 19].

Continuous and extensive application of Bti is required for efficient control of mosquitoes [20, 21]. This massive use of Bti imposes strong selective pressure [22]. However, the complex mode of action of the numerous synergistic endotoxins of Bti reduces the risk of emergence of resistance [23, 24]. Indeed, field studies consistently demonstrate the absence of resistance to Bti crystal [25–27], while resistance to individual toxins has been reported through laboratory selection procedures [28–30]. Although resistance to commercial Bti formulations has not been detected at the population level, some larvae can survive application of lethal doses of Bti and are thus considered tolerant. Whether Bti tolerant larvae suffer fitness costs at the adult stage, which could impact their vectorial capacity, is still not known. Neither the effect of a lethal concentration of Bti on the microbiota of tolerant larvae and the resulting adults has been investigated. Here we addressed these knowledge gaps by studying the long-term impact of Bti on the fitness and gut microbiota of tolerant larvae and their adult counterparts. We further compared the expression of nine selected immunity genes in the gut of Bti-tolerant versus larvae not exposed to Bti. Our results show that exposure to a lethal concentration of Bti during larval development alters gut microbiota composition in both larvae and adults, induces immune responses, and has carry-over effects on the fitness of adults derived from tolerant larvae.

## Methods

### Mosquito strain

We used three *Ae. albopictus* laboratory populations, namely Foshan (Fo), Tapachula (Tap), and Crema (Cr). Foshan is the *Ae. albopictus* reference strain, which originated in the early 1980s from field-collected eggs from the city of Foshan (China). Fo has been maintained and reared at the insectary of the University of Pavia since 2013, as previously described [31]. Tap and Cr were derived from field-collected eggs from Tapachula (Mexico) at the end of 2017 and from Crema (Italy) in 2018, respectively [31, 32]. Since establishment, these laboratory populations have been maintained in parallel under standard insectary conditions (28 °C, 12/12 h light/dark photoperiod, 70 ± 5% relative humidity). Larvae are reared in BugDorm plastic pans (19 × 19 × 6 cm) with controlled density (about 200 larvae in 1 L of water) to avoid competition for food resources, which are provided daily in the form of fish food (Tetra Goldfish Gold Color, Tetra Werke, Germany). Adults are maintained in insect-rearing cages (BugDorm 45 × 45 × 45 cm) and are fed with 0.2 g/ml sucrose on soaked cotton wool. Each colony is fed once a week with commercially defibrinated mutton blood (Biolife Italiana) for 90 min using a Hemotek blood-feeding system (Hemotek Ltd., Accrington, England).

### Assessment of larvicidal activity

We used VectoBac^®^ 12AS as the commercial formulation of *Bacillus thuringiensis* subsp. *israelensis* (strain AM65-52, fermentation solid and soluble) to compare the susceptibility of three *Ae. albopictus* laboratory populations, namely Fo, Tap, and Cr, to Bti infection. VectoBac® 12AS has a potency of 12,000 international toxin units (ITU) per milligram of formulation, which is equivalent to 1.3 × 10^9^ ITU/lr. Dose–response curves were generated against each of our three target populations, using standard laboratory bioassay methods for testing larval susceptibility [33]. For each laboratory population, dose–response assessment was based on three replicate experiments, each using batches of 120 third instar larvae. Briefly, before Bti application, larvae were starved for 3 h. Then Bti was applied at decreasing concentrations, starting from 6.395 × 10^3^ ITU/ml and halving each subsequent concentration such as 3.195 × 10^3^, 1.597 × 10^3^, 7.985 × 10^2^, 3.992 × 10^2^, 1.995 × 10^2^, and 9.981 × 10^1^ (ITU/ml). The number of dead larvae was counted 16–18 h after exposure to determine the lethal concentration 50 (LC_50_) and 80 (LC_80_), which correspond to the Bti concentration at which 50% or 80% of the exposed larvae died, respectively. A control consisting of 120 third instar larvae that were not exposed to Bti was included to assess the baseline larval mortality and ensure the validity of the dose–response assessment. If the mortality rate of the control group was above 20%, results were discarded, and the experiment was repeated.

### Identification of Bti-tolerant larvae

We used a total of 1800 third instar larvae, in six replicates of 300 larvae each, to identify Bti-tolerant larvae. Each replicate was exposed to 500 ml sterilized water containing Bti at the concentration of 7.036 × 10^–1^ ITU/ml (LC_80_). A total of 16 h after initial exposure, we collected surviving larvae, which were washed three times with sterilized water, carefully transferred to a new plastic container filled with sterilized water, and provided with finely ground fish food as their diet. These Bti-tolerant larvae were maintained under standard laboratory conditions to complete their development.

### Fitness assessment

Bti-tolerant larvae were raised to adulthood under standard insectary conditions, and 7 days post-emergence (DPE), 50 females were transferred to a new cage and offered a blood meal for 90 min, after which, fully engorged females were placed individually in cups. The number of engorged females served as a blood-feeding rate; 48 h after the blood meal, we provided water and egg papers for oviposition to each cup. We let mosquitoes oviposit for 72 h and counted the number of eggs laid by each female as a measure of female fecundity. Eggs from each female were hatched separately to assess fertility rates. The percentage of females that did not oviposit and those that produced non-fertile eggs were calculated as sterility and infertility, respectively. Adult longevity was measured by placing up to seven newly emerged mosquitoes of the same sex in each cup. Mosquitoes had ad libitum access to a 20% sucrose solution and their survival was daily monitored. A total number of 224 females and 304 males were used for survival analysis.

Fitness traits of the progeny of control and Bti-tolerant mosquitoes were assessed in six replicates as described elsewhere [34]. Briefly, batches of 100–120 eggs per replicate were hatched under standard insectary conditions and reared until adult emergence. In each replicate, emerging larvae, pupae, and adults were counted daily to assess egg hatchability, pupation rate, and percentage of adult emergence. We also measured larval developmental time (LDT) and pupal developmental time (PDT), the latter by counting emerging adults and subtracting LDT from the egg-to-adult developmental time. At emergence, adults were sexed and counted to measure sex ratio, egg-to-adult viability, and developmental speed for each sex [34].

Differences in blood feeding rate, fecundity, fertility, egg hatching rate, pupation rate, LDT, PDT, adult emergence rate, sex ratio, and egg-to-adult viability were tested using unpaired Student’s *t*-tests [35]. We compared mosquito survival using the Kaplan–Meier analysis and log-rank (Mantel–Cox) test [36]. Longevity data were used to extrapolate the median survival time and the hazard ratio, using the Mantel–Haenszel method [37].

### DNA extraction

We collected a total of 16 fourth instar larvae and 16 female adults that had survived Bti exposure, along with an equal number of control larvae and females that had not been exposed to Bti. Adult females were sampled 5 DPE between 10 am and 12 pm to avoid any bias related to the photoperiod. Before collection, individuals were surface sterilized by rinsing in a 2% sodium hypochlorite solution for 10 min, followed by surface disinfection for 5 min with 1 × phosphate buffered saline (PBS) solution and 70% ethanol for 2 min, and then washed twice with sterile water. Subsequently, the gut was dissected from larvae and adult females and DNA was extracted from single individuals. We used the Wizard® Genomic DNA Purification Kit (Promega) following manufacturer’s instructions for DNA extraction. DNA was resuspended in 20 μl of preheated DNase-free water and its concentration was determined using Nanodrop ND-1000 (Thermofisher).

### 16S metagenomic sequencing and analysis

DNA extracted from the guts of larvae and adults was shipped to Macrogen, Inc. for Illumina MiSeq 16S library preparation and sequencing. Libraries were generated following the standard Illumina protocol targeting the V3-V4 region of the 16S rRNA gene using primers 341F (5'-CCTACGGGNGGCWGCAG-3') and 805R (5'-GACTACHVGGGTATCTAATCC-3'). The libraries were sequenced on the MiSeq Illumina platform, resulting in 300 bp paired-end reads. Data analysis was carried out with the nf-core/ampliseq pipeline (version 2.5.0) deployed within Docker [38–40]. The pipeline includes a comprehensive workflow for the analysis of amplicon sequencing and the taxonomic assignment of 16S rRNA sequencing data. Briefly, we performed quality control of raw reads with FastQC [41]. We used Cutadapt [42] to trim forward and reverse reads on the basis of base quality assessment at 280 bp and 200 bp, respectively. We identified rRNA sequences with Barrnap [43] and used DADA2 for inference of amplicon sequence variants (ASVs) and filtering. ASVs were identified and classified by DADA2 [44] on the basis of the SILVA SSU non-redundant database (release 138) [45], retaining only sequences with a minimum frequency of 10 across all samples.

ASVs assigned to *Wolbachia* were included to test differential abundance between groups, but excluded for diversity analyses due to their preponderance in adult samples as previously done [32, 46]. The exclusion of *Wolbachia* resulted in unequal sequencing depths between adults and larvae samples; to account for this discrepancy, we performed rarefaction [47]. We used the nf-core ampliseq pipeline to perform random subsampling of reads in each sample, ten times for each sequencing depth, and generated rarefaction curves (Additional file 1: Fig. S1). We chose a rarefaction depth on the basis of the results of the rarefaction curves. As shown in Fig. S1, after excluding *Wolbachia*, 4000 sequences represent an exhaustive threshold for capturing the maximum observed features (i.e., OUTs) in each adult sample without losing bacterial diversity. As such, this depth was selected as the threshold and samples with less than 4000 sequences were discarded from diversity analysis. After setting the rarefaction depth threshold to 4000 sequences, we used QIIME2 [48] to calculate the absolute and relative abundance of ASVs within each sample and to determine the microbial community.

Microbial diversity was described in terms of alpha diversity, measured as the Shannon diversity index, which accounts for both the richness and evenness of species within a single sample, and beta diversity, evaluated using the Bray–Curtis index, which measures the differences in species composition and abundance between different samples [49]. Statistical differences in the overall alpha diversity between Bti-tolerant versus control larvae, adults emerging from Bti-tolerant larvae versus control adults, Bti-tolerant larvae versus their emerging adults, and larval versus adult controls were evaluated using the Kruskal–Wallis pairwise test. Statistical significance of comparisons of beta diversity among groups was assessed using the PerMANOVA pairwise test with 999 permutations. Differential abundance analysis of taxonomic units between groups was performed with ANCOM-BC2 [50] (q value < 0.05), implemented in the R package ANCOMBC (v.2.6.0) [51].

Bioinformatic analyses were performed on a 64-core Intel Xeon E5-2683 v4 Linux server and data visualization was performed with custom scripts implemented in R (version 4.2.3) [52].

### Expression analyses of selected genes

We dissected guts from 24 fourth instar larvae and 24 female adults collected 5 DPE from adults emerging from Bti-tolerant larvae; the same number of samples was collected for controls at the larval and adult stages. For each condition, we repeated dissection three times (i.e., three biological replicates), managing eight larval and eight adult samples each time. We extracted total RNA from individual guts using the TRIzol™ Plus RNA Purification Kit protocol (Invitrogen™) following the manufacturer’s instructions. Extracted RNA was treated with DNase (ThermoFisher) to remove genomic DNA and cDNA was synthesized using the GoScript™ Reverse Transcription Mix, Oligo(dT) according to the manufacturer’s instruction. cDNA was then used to quantify the transcriptional activity of nine selected genes: *Myd88* (AALC636_030222)*, Cactus* (AALFPA_063808), *Cecropin B* (AALFPA_043934), *Cecropin A* (AALF012131), *Dorsal* (AALFPA_074733), *Dicer 2* (AALFPA_062753), *Ago 2* (AALFPA_066143), *PPO* (AALFPA_056936), and *obp28* (AALFPA_044806). Primers were designed using the online tool for real-time PCR primer design of Primer-BLAST (https://www.ncbi.nlm.nih.gov/tools/primer-blast/) (Additional file 2: Table S1). Quantitative polymerase chain reaction (qPCR) was performed using the QuantiNova™ SYBR® Green PCR Kit (QuantiNova™) and the *Ae. albopictus* 60S ribosomal protein L34-like gene, *RpL34* (AALC636_026691) as housekeeping gene ​[53]​. Each qPCR reaction was run in a total volume of 20 μl, containing 10 μl SYBR Green (ThermoFisher), 4 μl of cDNA, 4 μl of H_2_O, and 1 μM of each of the forward and reverse primers. Cycling parameters were: 95 °C for 2 min followed by 40 cycles of 95 °C for 5 s, 60 °C for 30 s, followed by the melting curve generation.

The 2^−ΔΔCt^ method was used to calculate mRNA abundance for each gene of interest as previously shown [54], averaging the data of the three biological replicates; each individual sample was examined using technical duplicates. An unpaired *t*-test was used to test for significant differences in relative mRNA abundance between groups. We used GraphPad Prism (Version 9.1.1) for all statistical analyses.

## Results

We compared the susceptibility of three *Ae. albopictus* laboratory-populations, namely Fo, Tap, and Cr, to a Bti spore and crystal formulation on the basis of the AM65-52 strain. We plotted the percentage of mortality and log-transformed Bti concentration for each mosquito populations to obtain a dose–response curve and we used probit analysis to calculate the LC_50_ values (Additional file 3: Fig. S2). On the basis of the probit analysis (Table 1), Cr was found to be the most susceptible laboratory population, with LC_50_ and LC_80_ values of 314.2 (95% CI 256.4–411.9) and 645.06 (95% CI 487.4–802.6) ITU/ml, respectively. On this basis, we selected Cr to further investigate the carry-over effects of Bti exposure on the fitness, the microbiota, and the expression of immune genes in larvae surviving LC_80,_, hereafter called tolerant larvae, and adults emerging from these larvae.

**Table 1:** Probit analysis of Bti against different populations of *Ae. albopictus*

*Ae. albopictus* population	LC_50_ (ITU/ml)	LC_50_ 95% CI^a^	Slope	Slope 95% CI^a^	*χ*^2^
Crema	314.2	256.4 to 411.9	2.991	1.759 to 17.76	0.9674
Tapachula	455.2	341.7 to 622.4	2.501	1.467 to 4.801	0.9611
Foshan	719.9	589.6 to 923.6	2.669	1.619 to 6.138	0.9569

^a^95% confidence interval

### Bti exposure reduces emergence, longevity, and fecundity of adults derived from tolerant larvae

**Fig. 1:**
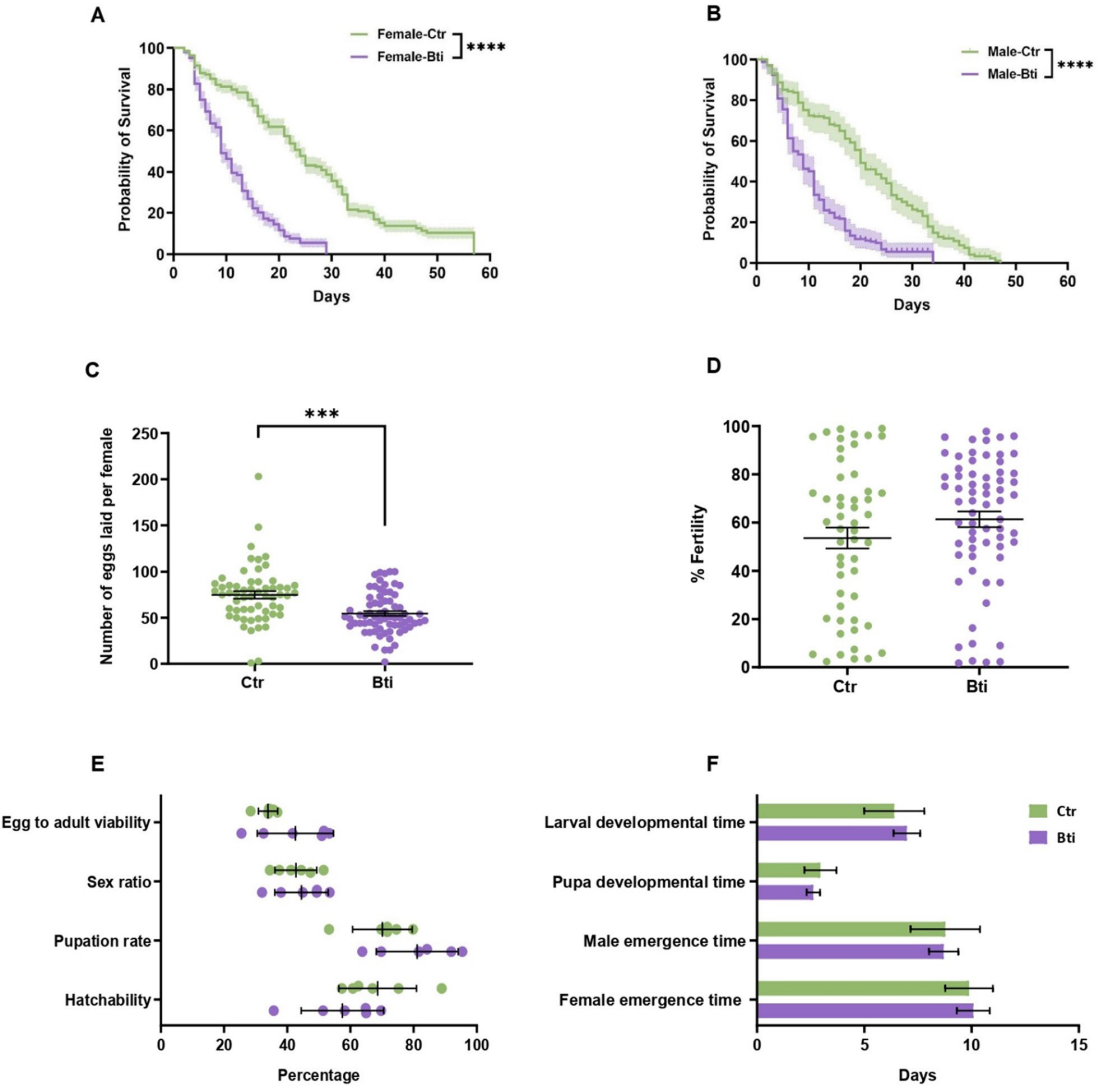
Fitness comparison between Bti-tolerant and control mosquitoes. Probability of survival of females (**A**) and males (**B**) of control and Bti-tolerant samples. Shaded areas in A and B represent 95% confidence intervals (CI). Differences in adult lifespan were tested via log-rank analyses. **C** Average number of eggs laid per female (fecundity). **D** Percentage of eggs deposited by each female that hatched (fertility). Each dot represents data for a female, differences in fecundity and fertility were compared using Student's *t*-test and error bars represent the 95% CI. **E** Fitness parameter of F1 progeny. **F** Developmental indices of F1 progeny. Comparison of the fitness parameters between Bti-tolerant and control samples was performed using Student’s *t*-test. Ns represents non-significant, *P-value < 0.05, **P-value < 0.01, ***P-value < 0.001, and ****P-value < 0.0001. In all the panels the controls are indicated in green and the Bti-tolerant samples are indicated in purple

We tracked the cost of Bti tolerance on adults emerging from tolerant larvae as well as the carry-over effect of Bti tolerance on the fitness of the subsequent generation. We followed the development of Bti-tolerant larvae and measured the percentage of adult emergence, the longevity of males and females, the blood feeding rate, and the fecundity and the fertility of females. We compared these fitness parameters with those of adults emerging from larvae not exposed to Bti, hereafter called controls. Only 45% (± 1.34) of Bti-tolerant larvae reached the adult stage, which is significantly lower than the value of 73% (± 5.94) observed in controls (*P*-value = 0.0153, Additional file 4: Table S2, Additional file 5: Fig. S3). Median survival time of females emerging from Bti-tolerant larvae was significantly shorter than that of females from control larvae, 10 (± 0.6) and 24 (± 1.2) days, respectively (Fig. 1A). The same trend was observed for males, with median survival time of males emerging from Bti-tolerant larvae being 9 (± 0.47) days with respect to 20 (± 0.96) days of males from control larvae (Fig. 1B). In addition, 1-week-old females exposed to a blood meal showed comparable blood-feeding rates when emerging from Bti-tolerant (37.39% ± 5.68) or control larvae (37.65% ± 4.28) (Additional file 5: Fig. S3). Additionally, there was also no significant difference in the percentage of sterile females (5.53% ± 2.3 and 7.07% ± 4.97 from either Bti-tolerant or control larvae, Additional file 5: Fig. S3) or the percentage of infertile eggs (5.185% ± 3.47 and 12.33% ± 5.173 from females derived from either Bti-tolerant or control larvae, Additional file 5: Fig. S3). However, females emerging from Bti-tolerant larvae showed significantly lower fecundity with respect to those from control larvae (54.0 ± 2.77 and 74.33 ± 4.01 in females from Bti-tolerant versus control larvae, respectively, Fig. 1C). Despite having laid less eggs, the fertility of adults emerged from Bti-tolerant larvae was not significantly different than that of control mosquitoes (61.32% ± 3.28 and 53.54% ± 4.34 in females from Bti-tolerant or control larvae, Fig. 1D, Additional file 4: Table S2).

We further followed the development of eggs laid by adults emerging from Bti-tolerant versus control larvae and observed no differences in the percentage of egg viability, LTD, PDT, pupation rate, egg-to-adult viability, sex ratio, and developmental speed of males and females; values for each of these parameters and relative statistics are shown in Fig. 1E, F and Additional file 4: Table S2. Overall, these results highlight that Bti exposure influences the emergence, longevity, and fecundity of adults from Bti-tolerant larvae, but these effects are not passed on to the following generation.

### Bti exposure alters the richness of several bacterial genera and increases microbial diversity

We continued the analyses of the effects of Bti on the microbiota of Bti-tolerant larvae and emerging adults using 16S barcode sequencing [55]. We compared the gut microbiota diversity, composition, and abundance across all our experimental groups.

**Fig. 2:**
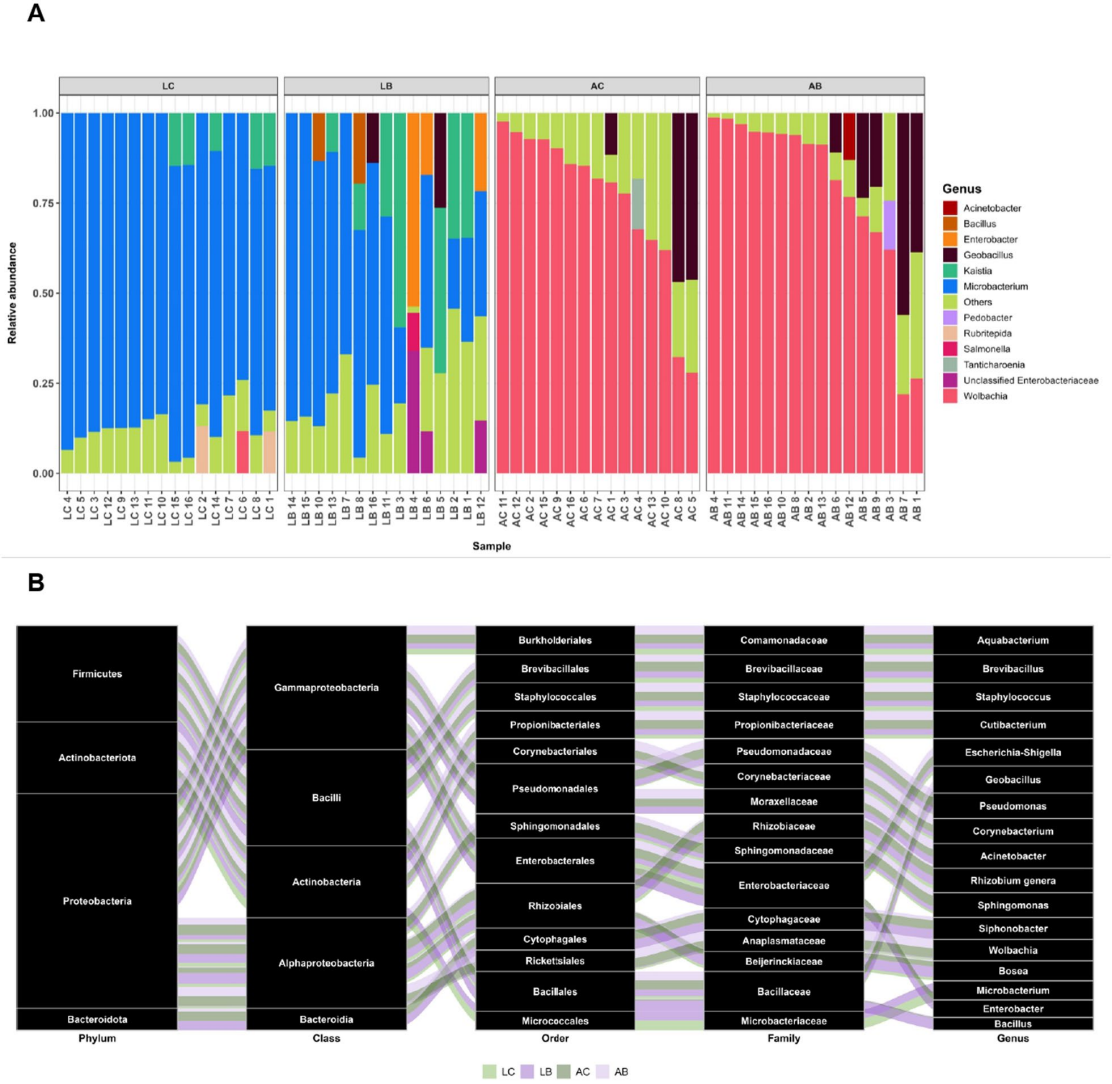
Changes in the relative abundance of components of the gut microbiota of Bti-tolerant mosquitoes versus their controls. **A** The relative abundance bar plot of bacterial composition at the genus taxonomic level. Each bar represents the relative abundance assigned to a given bacterial genus for each individual gut. Only genera with a relative abundance greater than 10% in each individual gut are represented; genera with a relative abundance below 10% are grouped into “Others.” **B** Alluvial plot showing the prevalence of differentially abundant taxa from phylum to genus in the gut of Bti-tolerant larvae (LB), their controls (LC), and in the gut of adults emerging from Bti-tolerant larvae (AB) and their controls (AC). Each taxonomic level is represented by a black square and it is organized in ascending order on the basis of the most abundant genera across all experimental groups. The most abundant genus (i.e., *Acquabacterium*) appears at the top of the last column on the right, while the least abundant genus (i.e., *Bacillus*) is listed at the bottom. The width of each line corresponds to the abundance of the taxa in LB, LC, AB, and AC samples. Green and purple colors represent control and Bti-tolerant samples, respectively. Light and dark shades of green and purple refer to larval and adult samples, respectively

**Fig. 3:**
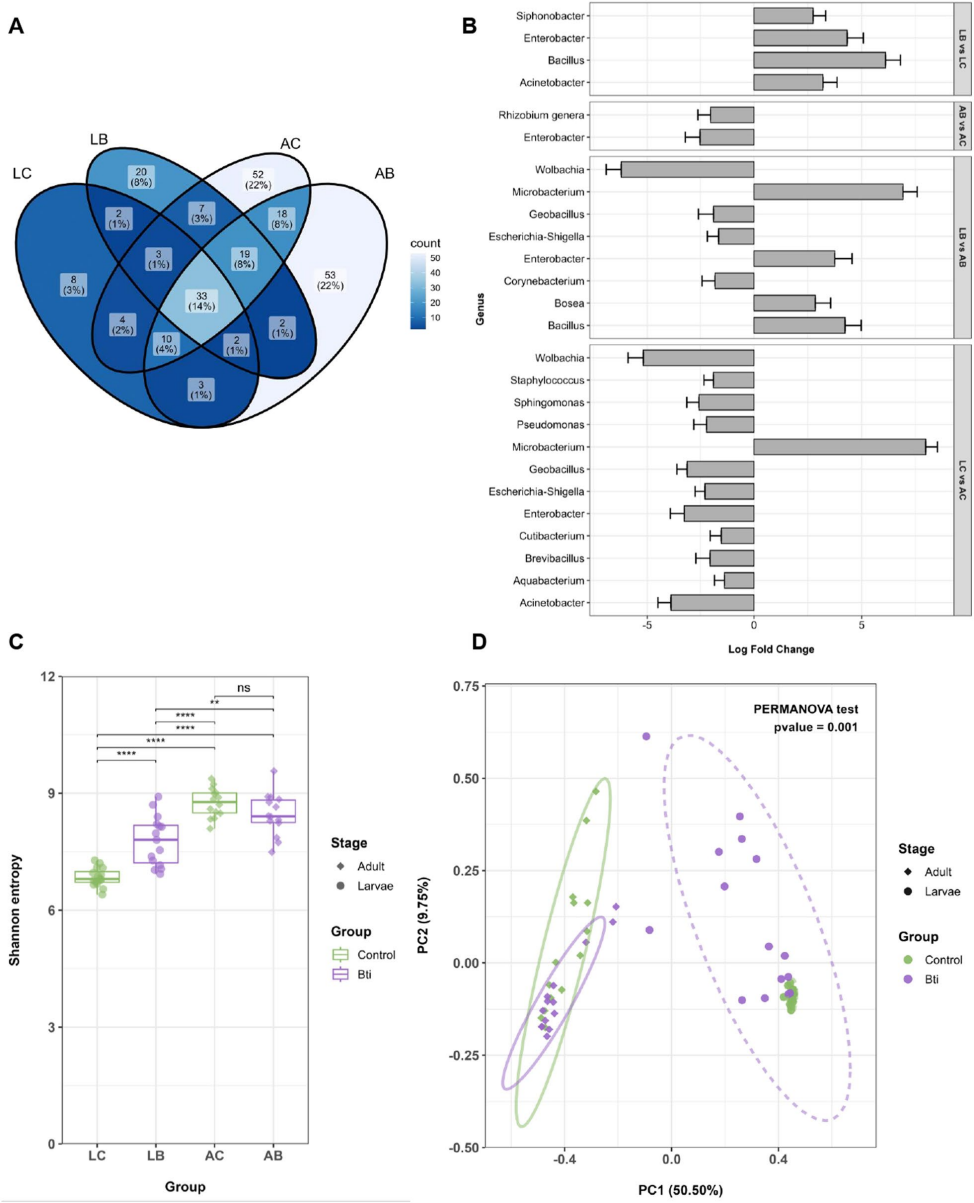
Changes in the composition of gut microbiota of Bti-tolerant mosquitoes versus control samples. **A** Venn diagram showing the number (and percentage) of bacterial genera that are either in common or unique to Bti-tolerant larvae (LB), their emerging adults (AB), control larvae (LC), and control adults (AC). **B** Differential abundance analysis bar plot showing the pairwise comparisons of fold changes of bacterial genera in common between LB versus LC, AB versus AC, LB versus AB, and LC versus AC. **C** Alpha diversity based on the Shannon index. **D** Principal coordinate analysis (PCoA) based on the Bray–Curtis metric of the microbiota diversity and richness across samples. Ns represents non-significant, *P-value < 0.05, **P-value < 0.01, ***P-value < 0.001, and ****P-value < 0.0001

We generated and sequenced a total of 64 libraries, 16 from fourth instar Bti-tolerant larvae (LB), 16 from fourth instar control larvae (LC), 16 from adult females emerging from Bti-tolerant larvae (AB), and 16 from adult females from control larvae (AC). One LB and one AC library were discarded after quality control assessment. Between 189,508 and 361,410 sequencing reads were obtained across our remaining 62 samples (Additional file 6: Table S3). After DADA2 filtering, the number of ASVs per sample ranged between 55,433 and 154,448, resulting in a total of 154 bacterial families and 291 genera (Additional file 7: Table S4). Taxonomic classification revealed that the most dominant genera in LB samples are *Microbacterium*, *Kaistia*, *Enterobacter*, *Bacillus*, *Geobacillus*, and *Miniimonas*. The predominant genera of LC samples are *Microbacterium*, *Kaistia*, *Rubritepida*, *Wolbachia*, *Miniimonas*, and *Geobacillus* (Fig. 2A, Additional file 8: Table S5). We also observed significant changes in the composition of microbiota across experimental groups (Fig. 2B). A total of 40 genera were shared between LB and LC samples, excluding unclassified taxa (Fig. 3A, Additional file 8: Table S5), but shared genera had different relative abundances (Fig. 3B). For instance, in LB versus LC samples, we observed a significant increase in the relative abundance of *Siphonobacter*, *Enterobacter*, *Bacillus*, and *Acinetobacter* (Fig. 3B).

The observed changes in the richness and diversity of the microbiota of LB and LC samples are supported by the results of the alpha and beta diversity metrics (Fig. 3 C and D, Additional file 9: Table S6). Beta diversity metrics showed that while LC samples clustered closely together, indicating a high degree of similarity in their gut microbiota composition, LB samples were dispersed, reflecting greater variability among individuals (Fig. 3D).

The alpha diversity, based on the Shannon index, indicates no significant difference in the gut microbiota diversity between AB and AC samples (Fig. 3C). The microbiota of adults, from both AB and AC samples, was dominated by *Wolbachia* (Additional file 8: Table S5) with 78.79 ± 24.31% and 75.6 ± 21.45% of ASVs linkable to *Wolbachia* in AB and AC samples, respectively. Excluding *Wolbachia*, the most represented bacterial genera in AC samples were *Geobacillus*, *Tanticharoenia*, *Enterobater*, *Klebsiella*, *Escherichia*-*Shigella*, and *Acitenobacter* (Additional file 10: Fig. S4). The dominant genera of AB samples were *Geobacillus*, *Staphylococcus*, *Pedobacter*, *Escherichia*-*Shigella*, and *Acitenobacter* (Additional file 8: Table S5). A total of 80 genera were shared between AC and AB samples (Fig. 3A, Additional file 11: Table S7), among them, *Enterobacter* and *Allorhizobium*-*Neorhizobium*-*Pararhizobium*-*Rhizobium* had significantly lower relative abundance in AB versus AC (Fig. 3B, Additional file 12: Table S8). Pairwise PerMANOVA analysis based on the Bray–Curtis distance metric revealed significant differences in the composition of the bacterial community between AB and AC samples and also LC versus LB samples (Fig. 3D, Additional file 9: Table S6). This result is corroborated by PCoA analysis, which showed that, excluding *Wolbachia*, AC and AB samples cluster separately from LC and LB samples and manifest high intra-condition variability. Moreover, the absence of some genera (i.e., *Salmonellai*, *Klebsiella*, *Vulcaniibacterium*, *Siphonobacter*, *Mesorhizobium*, and *Clostridium*) in AB samples suggests that the effect of Bti extends beyond the larval stage (Additional file 7: Table S4).

### Bti exposure alters the richness of the core microbiota

We looked at the intersections of bacterial genera found in our samples to identify the components of the microbiota that are conserved across all samples and their richness (Additional file 11: Table S7). We observed that 33 genera were shared across all samples, including larvae and adults, suggesting they are the “core microbiota.” Among these, *Microbacterium*, *Wolbachia*, *Geobacillus*, *Enterobacter*, *Acinetobacter*, *Escherichia-Shigella*, *Methylobacterium*, and *Aquabacterium* were the most abundant. Despite the presence of these bacterial genera being conserved across all samples, their relative abundances were different between Bti-tolerant and control samples. For instance, *Enterobacter* had a significantly higher abundance in Bti-tolerant larvae versus adults, whereas the opposite was true for LC versus AC (Fig. 3B, Additional file 12: Table S8). The relative abundance of *Bacillus* and *Bosea* genera were significantly higher in LB versus AB, suggesting Bti-dependent changes in core microbiota richness. We further observed that LB and AB samples share two bacterial genera, namely *Blautia* and *Coxiella*, which were absent in control samples and their abundance did not differ between the larval and adult stages. LC and AC samples shared three bacterial genera (*Caldimonas, Roseomonas*, and *Rothia*), which were absent in both LB and AB samples.

As expected, there were changes in the overall gut microbiota composition from larvae to adult. Interestingly, the relative abundance of *Enterobacter* was significantly higher in LB versus AB (6.8-fold), while the relative abundance of this bacterium decreased 5.6-fold in LC versus AC samples (Fig. 3B, Additional file 12: Table S8). Overall, we saw less changes in the relative abundances of bacteria and more shared microbiota between LB and AB versus LC and AC, suggesting that Bti-tolerant larvae contributed more to the microbiota composition of emerging adults with respect to control larvae.

### Bti exposure elicits immune responses in the gut of *Ae. albopictus*

**Fig. 4:**
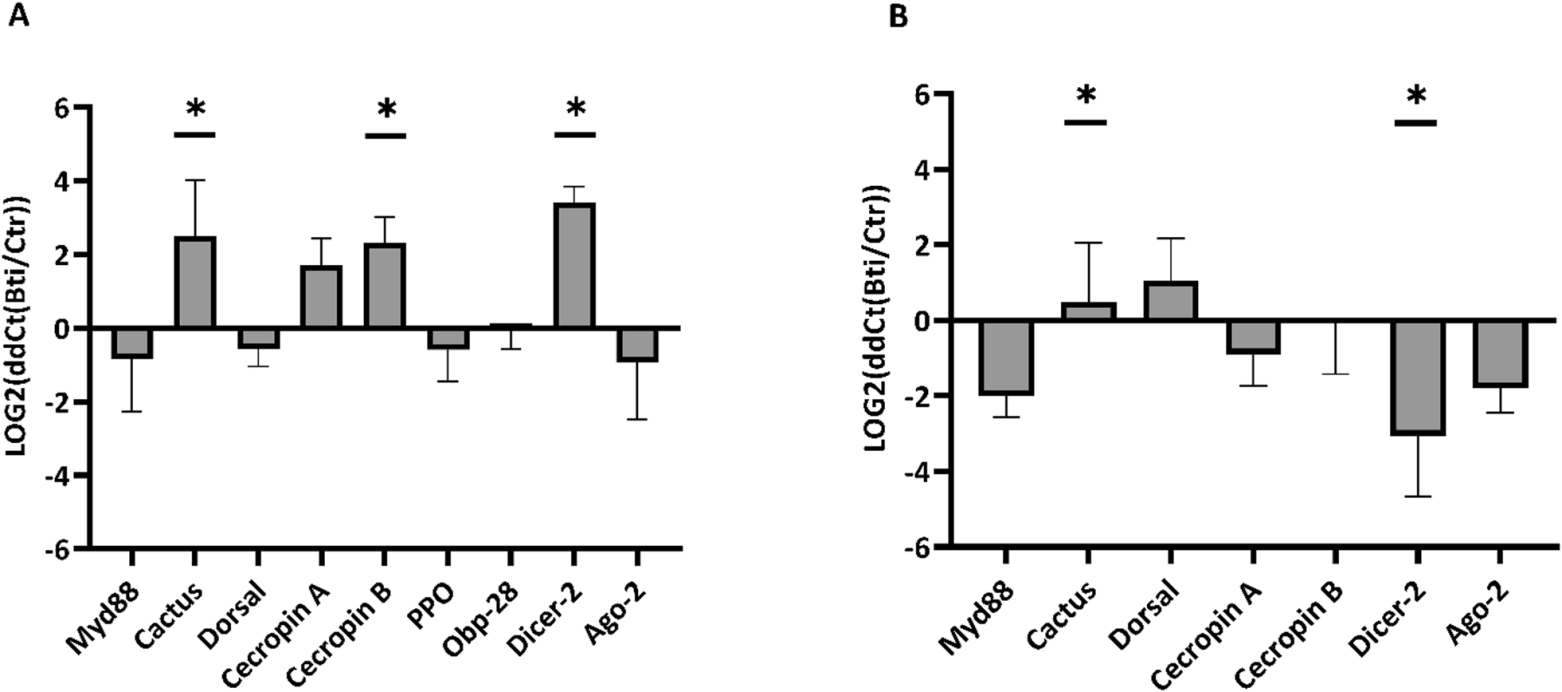
Relative expression of nine immunity genes in Bti-tolerant versus control larvae and adults. The expression of each gene in the gut of Bti-tolerant larvae (**A**) and adults (**B**) was compared with its control. Each bar represents the mean (± SD) of three biological replicates. *P-value < 0.05

We compared the transcriptional activity of *myd88, cactus, dorsal*, and *cecropinA*, which represent modulators and/or effectors of the Toll pathway [56, 57]; *cecropinB*, the antimicrobial peptide that is synthesized upon activation of the IMD pathway [58]; *PPO* and *obp28*, which contribute to pathogen melanization [59, 60], and *dicer2* and *ago2*, whose activity results in synthesis of small interfering RNAs (sRNA) [61] in larvae. To verify the carry-over effects of Bti exposure on adults derived from Bti-tolerant larvae, we further studied the expression of all the mentioned immune genes in adults, except for *PPO* and *obp28.* We exclude *obp28* since its immune function has been shown only in larvae and *PPO* is activated only in the presence of a pathogen [60, 62]. Figure 4 summarizes the relative expression of each of these genes in Bti-tolerant larvae and adult versus their control counterparts, after normalization of their expression to that of the housekeeping gene *rpl34*. Significant differential expression was observed in *cactus*, *cecropinB*, and *dicer2* genes in larvae, which had 2.5-, 2.3-, and 3.4-fold higher expression in Bti-tolerant versus control larvae, respectively (Fig. 4A). In females emerging from Bti-tolerant larvae, the expression of *cactus* was 0.5-fold higher and *dicer2* was expressed threefold less compared with control females (Fig. 4B).

## Discussion

Bti application is a safe, eco-friendly, and effective method to control mosquito larval populations, that, to date, has not resulted in emergence of resistance [63]. Despite the absence of resistance at the population level, some larvae exposed to lethal concentrations of Bti survive. These larvae are defined as being Bti-tolerant. Our results showed that Bti-tolerant larvae suffer fitness costs, which are passed to the adult stage, but not to the following generation. We also observed significant changes in the composition of the gut microbiota both in Bti-tolerant larvae and the derived adults.

In mosquitoes, larval and adult stages live in different ecological niches and the larval developmental conditions can influence adult life history traits and vectorial capacity. These conditions include both biotic and abiotic factors such as temperature, diet, and habitat microbial community [64–68]. For instance, application of sublethal doses of Bti to larval breeding sites resulted in adults that were smaller and shorter lived, and females with a lower blood feeding rate and fecundity, traits which can impact vectorial capacity [69–71]. In our study, survival after exposure to Bti lethal doses resulted in larvae giving rise to adults that suffered fitness costs in terms of reduced emergence rate, shortened lifespan, and decreased fecundity. Despite the observed costs in *Ae. albopictus* adults derived from Bti-tolerant larvae, the fitness parameters of the subsequent generation were not affected highlighting the lack of long-term indirect effects of Bti-based insecticides.

We also showed significant changes in the gut microbiota of Bti-tolerant larvae and emerging adults, which align with previous data showing significant alterations in the microbial communities of *Ae. aegypti* and *Cx. pipiens* mosquitoes exposed to different concentrations of Bti [17, 18]. Consistent with our findings, *Cx. pipiens* larvae exposed to Bti showed higher alpha diversity in their gut microbiota compared with the control [18]. In contrast, the microbiota of Bti-tolerant *Ae. aegypti* larvae exhibited lower diversity compared to non-tolerant individuals [17]. Nevertheless, the microbiota of Bti-tolerant *Ae. aegypti* larvae showed high inter-individual variability, consistent with our findings [17]. In general, larval microbiota appears to be shaped by the breeding site, since strong similarities in the composition of larval microbiota were found in larvae that developed in the same site [72]. Accordingly, we observed low inter-individual variability among LC samples on the basis of beta diversity analysis. However, LB samples were more dispersed, as seen in *Ae. aegypti* [17], further emphasizing the role of Bti in modulating the composition of larval microbiota.

Metamorphosis and molting are known to reduce the richness of the gut microbiota of adult *Anopheles*, *Culex*, and *Aedes* mosquitoes [73], which we also observed. We further saw changes in the composition of the gut microbiota between AB and AC samples and changes in the relative abundance of components of their core microbiota. Altering the relative abundance of the core microbiota may lead to restructuring of the essential gut bacterial communities impacting mosquito fitness and nutrient assimilation [74–79]. Thus, the fitness changes observed in AB females may be partly linked to alterations in their microbiota composition, including changes in the relative abundance of their core microbiota. Additional functional studies, which are beyond the scope of this work, are necessary to draw solid conclusions regarding the role(s) of specific microbes on mosquito traits.

The disruption of host cell membranes by Bt toxins and the invasion of Bt spores and gut-residing bacteria into the insect hemolymph trigger humoral and cellular immune defenses in the host. These defenses include encapsulation, phagocytosis, melanization, increased lysozyme activity, prophenoloxidase activation cascade, and production of antimicrobial peptides regulated by the IMD (immune deficiency) and Toll signaling pathways, among others [79–82]. Host immune responses activated by Bt can counteract its toxic effects and may contribute to the development of tolerance or resistance in exposed populations [79]. Bti infection was shown to increase the relative expression of genes associated with the Toll and IMD signaling pathways in larvae of *Cx. pipiens* and *Ae. aegypti* [18, 29]. Similarly, we observed changes in the relative expression of *cactus*, *cecropin B*, and *dicer2* in Bti-tolerant larvae. We further observed that the expression of *cactus* is higher in adults derived from Bti-tolerant larvae compared with controls, while the expression of *dicer-2* was reduced in adults from Bti-tolerant larvae, suggesting long-lasting changes from Bti exposure. In mosquitoes, changes in immune gene expression in Bti-tolerant populations have been linked to altered viral susceptibility [63]. For instance, prolonged exposure to Bti has been shown to reduce the expression genes belonging to the Toll pathway and of antimicrobial peptides in *Ae. aegypti*, and sublethal exposure to Bti increases adult susceptibility to DENV, but not CHIKV [63].

The immune system plays a crucial role in maintaining gut microbiota homeostasis [13], but Bt infection was shown to alter the abundance and composition of the gut microbiota, which, together with the elevated immune response, can influence the overall health of mosquitoes and possibly their ability to transmit diseases [17, 18, 83, 84]. Although Bti triggers immune responses in the insect gut [13, 18], altered microbiota can also elicit immune responses [17]. For instance, we observed overexpression of *cecropin,* which is generally more active against Gram-negative than Gram-positive bacteria [82]. This might correlate with the enrichment of Gram-negative bacteria such as *Enterobacter*, *Acinetobacter*, and *Siphonobacter* in LB samples. Overall, these results underline the intricate relationship among Bti, gut microbiota, and immunity generating intricate back-interactions among each other and mosquito fitness.

## Conclusions

Bti exposure exerts a complex influence on *Ae. albopictus* by controlling the population size of mosquitoes and reducing the fitness of adults emerging from tolerant larvae. We also show that Bti alters the gut microbiota of tolerant larvae and their emerging adults, including changes in the richness of the core microbiota, and induces immune responses in the larval and adult gut. These immediate effects negatively alter traits that directly or indirectly impact mosquito vectorial capacity [84], which further supports the use of Bti in control strategies. Additionally, the fitness parameters of the subsequent generation of mosquitoes remained unaffected, suggesting a lack of long-term indirect effects of Bti-based insecticides on *Ae. albopictus*.

## Supplementary Information

Additional file 1: Figure S1. Rarefaction analyses to ensure the unbiased comparisons of species richness and diversity metrics across all samples. The rarefaction depth of 4000 sequences was chosen on the basis of the rarefaction curves. Samples with a rarefaction depth lower than this threshold were discarded from diversity analysis.

Additional file 2: Table S1. List of primers for gene expression analysis.

Additional file 3: Figure S2. Susceptibility of three different laboratory populations of *Aedes albopictus* to Bti-based bio-insecticide. A Dose–response curve of Foshan, Tapachula, and Crema populations exposed to different concentrations of Bti. Dashed line refers to the log dose of the LC_50_. B Probit analysis of Crema strain. The plot illustrates the transformation of the dose–response curve into the linear regression between the log dose and probit of mortality on the basis of the Finney’s table [85]. The dashed line refers to the log dose of the LC_80_.

Additional file 4: Table S2. Comparison of values of life-table parameters in tolerant and control larvae, emerging adults, and the progeny from these adults (F1).

Additional file 5: Figure S3. Fitness comparisons between Bti-tolerant and control adults. Percentage of adult emergence (A), blood feeding rate (B), sterile female (C), and non-fertile eggs (D) of Bti and control groups. Green and purple colors represent control samples and Bti-tolerant samples, respectively. Comparison of the fitness parameters between Bti-tolerant and control samples was performed using Student’s *t*-test. *Represents *P*-value < 0.05.

Additional file 6: Table S3. Summary of number of sequences with DADA2 filtering.

Additional file 7: Table S4. Average relative abundance of sequences assigned to a given bacterial taxon at genus level.

Additional file 8: Table S5. Top 50 most abundant genera based on relative abundance.

Additional file 9: Table S6. Comparison of diversity metrics of the gut microbiota.

Additional file 10: Figure S4. Bar plot of the relative abundances of microbial community at the rank of phylum (A), family (B), and genus (C) in the gut of Bti-tolerant larvae and their derived adults and respective controls. Each bar in panels A and B represents the most abundant bacterial phylum and family, respectively, for each experimental group. Each bar in panel C shows the relative abundance assigned to a given bacterial genus, excluding *Wolbachia*, for each individual gut.

Additional file 11: Table S7. Common bacterial genera between groups and their average relative abundance.

Additional file 12: Table S8. Statistical analysis of differentially abundant genus in each experimental group.

## Data Availability

No datasets were generated or analyzed during the current study.

## Abbreviations

Bti: *Bacillus thuringiensis* Subsp. *Israelensis*
ITU: International toxin units
LC50: Lethal concentration 50
LC80: Lethal concentration 80
DPE: Days post-emergence
LDT: Larval developmental time
PDT: Pupal developmental time
PBS: Phosphate buffered saline
ASV: Amplicon sequence variants
LB: Bti-tolerant larvae
LC: Control larvae
AB: Adult females emerging from Bti-tolerant larvae
AC: Adult females from control larvae

## References

[1] WHO. Vector-borne diseases. 2020. https://www.who.int/news-room/factsheets/detail/vector-borne-diseases. Accessed 2 Mar 2020.

[2] Delatte H, Bagny L, Brengue C, Bouetard A, Paupy C, Fontenille D. The invaders: Phylogeography of dengue and chikungunya viruses *Aedes* vectors, on the South West islands of the Indian Ocean. Infect Genetics Evol. 2011;11:7. 10.1016/j.meegid.2011.07.016 21827872

[3] Bonizzoni M, Gasperi G, Chen X, James AA. The invasive mosquito species *Aedes albopictus*: current knowledge and future perspectives. Trends Parasitol. 2013;29:9. 10.1016/j.pt.2013.07.003 PMC3777778 23916878

[4] Londono-Renteria B, Troupin A, Colpitts TM. Arboviruses and potential transmission blocking vaccines. Parasites Vectors. 2016;9:516. 27664127 10.1186/s13071-016-1802-0 PMC5035468

[5] Sulaiman S, Pawanchee ZA, Wahab A, Jamal J, Sohadi AR. Field evaluation of Vectobac G, Vectobac 12AS and Bactimos WP against the dengue vector *Aedes albopictus* in tires. J Vector Ecol. 1997;22:2. 9491362

[6] Tan AW, Loke SR, Benjamin S, Lee HL, Chooi KH, Sofian-Azirun M. Spray application of *Bacillus**thuringiensis* israelensis (Bti strain AM65–52) against *Aedes**aegypti* (L.) and *Ae*. *albopictus* Skuse populations and impact on dengue transmission in a dengue endemic residential site in Malaysia, Southeast Asian. J Trop Med Public Health. 2012;43:2. 23082582

[7] Ben-Dov E. *Bacillus**thuringiensis* subsp. *israelensis* and its dipteran-specific toxins. Toxins. 2014;6:4. 10.3390/toxins6041222 PMC4014730 24686769

[8] Soberón M, Fernández LE, Pérez C, Gill SS, Bravo A. Mode of action of mosquitocidal *Bacillus thuringiensis* toxins. Toxicon. 2007;49:5. 10.1016/j.toxicon.2006.11.008 17145072

[9] Likitvivatanavong S, Chen J, Evans AM, Bravo A, Soberon M, Gill SS. Multiple receptors as targets of cry toxins in mosquitoes. J Agric Food Chem. 2011;59:7. 10.1021/jf1036189 PMC3686494 21210704

[10] Pardo-López L, Soberón M, Bravo A. *Bacillus thuringiensis* insecticidal three-domain Cry toxins: mode of action, insect resistance and consequences for crop protection. FEMS Microbiol Rev. 2013;37:1. 22540421 10.1111/j.1574-6976.2012.00341.x

[11] Schnepf E, Crickmore N, Van Rie J, Lereclus D, Baum J, Feitelson J, et al.*Bacillus thuringiensis* and its pesticidal crystal proteins. Microbiol Mol Biol Rev. 1998;62:3. 10.1128/mmbr.62.3.775-806.1998 PMC98934 9729609

[12] Wang Y, Gilbreath TM, Kukutla P, Yan G, Xu J. Dynamic gut microbiome across life history of the malaria mosquito *anopheles gambiae* in Kenya. PLoS ONE. 2011;6:9. 10.1371/journal.pone.0024767 PMC3177825 21957459

[13] Li S, de Mandal S, Xu X, Jin F. The tripartite interaction of host immunity-*Bacillus thuringiensis* infection-gut microbiota. Toxins. 2020;12:8. 10.3390/toxins12080514 PMC7472377 32806491

[14] Cherif A, Rezgui W, Raddadi N, Daffonchio D, Boudabous A. Characterization and partial purification of entomocin 110, a newly identified bacteriocin from *Bacillus**thuringiensis* subsp. Entomocidus HD110. Microbiol Res. 2008;163:6. 10.1016/j.micres.2006.10.005 19216106

[15] Patil CD, Borase HP, Salunke BK, Patil SV. Alteration in *Bacillus thuringiensis* toxicity by curing gut flora: novel approach for mosquito resistance management. Parasitol Res. 2013;112:9. 10.1007/s00436-013-3507-z 23820604

[16] Regode V, Kuruba S, Mohammad AS, Sharma HC. Isolation and characterization of gut bacterial proteases involved in inducing pathogenicity of *Bacillus thuringiensis* toxin in Cotton Bollworm, *Helicoverpa**armigera*. Front Microbiol. 2016;7:1567. 27766093 10.3389/fmicb.2016.01567 PMC5052264

[17] Tetreau G, Grizard S, Patil CD, Tran FH, Van Tran V, Stalinski R, et al. Bacterial microbiota of *Aedes aegypti* mosquito larvae is altered by intoxication with *Bacillus thuringiensis israelensis*. Parasit Vectors. 2018;11:1. 29499735 10.1186/s13071-018-2741-8 PMC5834902

[18] Zhang R, Liu W, Zhang Q, Zhang X, Zhang Z. Microbiota and transcriptome changes of *Culex pipiens* pallens larvae exposed to *Bacillus thuringiensis israelensis*. Sci Rep. 2021;11:1. 34642414 10.1038/s41598-021-99733-8 PMC8511237

[19] Broderick NA, Raffa KF, Handelsman J. Midgut bacteria required for *Bacillus thuringiensis* insecticidal activity. Proc Natl Acad Sci USA. 2006;103:41. 10.1073/pnas.0604865103 PMC1622799 17005725

[20] Ferreira LM, Silva-Filha MH. Bacterial larvicides for vector control: mode of action of toxins and implications for resistance. Biocontrol Sci Technol. 2013;23:10.

[21] Brühl CA, Després L, Frör O, Patil CD, Poulin B, Tetreau G, et al. Environmental and socioeconomic effects of mosquito control in Europe using the biocide *Bacillus**thuringiensis* subsp. *israelensis* (Bti). Sci Total Environ. 2020;724:137800. 32249002 10.1016/j.scitotenv.2020.137800

[22] Lacey LA. *Bacillus thuringiensis* serovariety *Israelensis* and *bacillus sphaericus* for mosquito control. J Am Mosq Control Assoc. 2007;23:133–63. 17853604 10.2987/8756-971X(2007)23[133:BTSIAB]2.0.CO;2

[23] Pérez C, Fernandez LE, Sun J, Folch JL, Gill SS, Soberón M, et al. *Bacillus**thuringiensis* subsp. israelensis Cyt1Aa synergizes Cry11Aa toxin by functioning as a membrane-bound receptor. Proc Natl Acad Sci USA. 2005;102:51. 16339907 10.1073/pnas.0505494102 PMC1317914

[24] Poncet S, Delécluse A, Klier A, Rapoport G. Evaluation of synergistic interactions among the CryIVA, CryIVB, and CryIVD toxic components of *Bacillus**thuringiensis* subsp. *israelensis* crystals. J Invertebr Pathol. 1995;6:2.

[25] Carvalho KDS, Crespo MM, Araújo AP, Da Silva RS, De Melo-Santos MAV, De Oliveira CMF, et al. Long-term exposure of *Aedes**aegypti* to *Bacillus**thuringiensis* svar. *Israelensis* did not involve altered susceptibility to this microbial larvicide or to other control agents. Parasit Vectors. 2018;1:1. 10.1186/s13071-018-3246-1 PMC6311009 30594214

[26] Araújo AP, Araujo Diniz DF, Helvecio E, De Barros RA, De Oliveira CMF, Ayres CFJ, et al. The susceptibility of *Aedes aegypti* populations displaying temephos resistance to *Bacillus thuringiensis israelensis*: a basis for management. Parasit Vectors. 2013;6:1. 24499507 10.1186/1756-3305-6-297 PMC3852962

[27] Becker N, Ludwig M, Su T. Lack of resistance in aedes vexans field populations after 36 years of *bacillus**thuringiensis* subsp. *israelensis* applications in the upper rhine valley, Germany. J Am Mosq Control Assoc. 2018;34:2. 10.2987/17-6694.1 31442151

[28] Stalinski R, Laporte F, Tetreau G, Després L. Receptors are affected by selection with each *Bacillus thuringiensis israelensis* Cry toxin but not with the full Bti mixture in *Aedes aegypti*. Infect Genetics Evol. 2016;44:218–27. 27418233 10.1016/j.meegid.2016.07.009

[29] Georghiou GP, Wirth MC. Influence of exposure to single versus multiple toxins of *Bacillus**thuringiensis* subsp. *israelensis* on development of resistance in the mosquito *Culex**quinquefasciatus* (Diptera: Culicidae). Appl Environ Microbiol. 1997;63:3. 10.1128/aem.63.3.1095-1101.1997 PMC1389136 16535542

[30] Tetreau G, Stalinski R, David JP, Després L. Monitoring resistance to *Bacillus**thuringiensis* subsp. *israelensis* in the field by performing bioassays with each Cry toxin separately. Mem Inst Oswaldo Cruz. 2013;10:7. 10.1590/0074-0276130155 PMC3970644 24037105

[31] Crava CM, Varghese FS, Pischedda E, Halbach R, Palatini U, Marconcini M, et al. Population genomics in the arboviral vector *Aedes aegypti* reveals the genomic architecture and evolution of endogenous viral elements. Mol Ecol. 2021;30:7. 10.1111/mec.15798 PMC8048955 33432714

[32] Scolari F, Sandionigi A, Carlassara M, Bruno A, Casiraghi M, Bonizzoni M. Exploring changes in the microbiota of *Aedes albopictus*: comparison among breeding site water, larvae, and adults. Front Microbiol. 2021;12:624170. 33584626 10.3389/fmicb.2021.624170 PMC7876458

[33] WHO. Guidelines for laboratory and field testing of mosquito larvicides. World Health Organization. 2005. https://www.who.int/publications/i/item/WHO-CDSWHOPES-GCDPP-2005.13. Accessed 24 May 2005.

[34] Carlassara M, Khorramnejad A, Oker H, Bahrami R, Lozada-Chávez AN, Mancini MV, et al. Population-specific responses to developmental temperature in the arboviral vector *Aedes albopictus*: implications for climate change. Glob Chang Biol. 2024;30:3. 10.1111/gcb.17226 38454541

[35] Lee S, Lee DK. What is the proper way to apply the multiple comparison test? Korean J Anesthesiol. 2018;71:5. 10.4097/kja.d.18.00242 PMC6193594 30157585

[36] Kishore J, Goel M, Khanna P. Understanding survival analysis: Kaplan-Meier estimate. Int J Ayurveda Res. 2010;1:4. 21455458 10.4103/0974-7788.76794 PMC3059453

[37] Muenz LR, Green SB, Byar DP. Applications of the Mantel-Haenszel statistic to the comparison of survival distributions. Biometrics. 1977;33:4. 338042

[38] Straub D, Blackwell N, Langarica-Fuentes A, Peltzer A, Nahnsen S, Kleindienst S. Interpretations of environmental microbial community studies are biased by the selected 16S rRNA (Gene) amplicon sequencing pipeline. Front Microbiol. 2020;11:550420. 33193131 10.3389/fmicb.2020.550420 PMC7645116

[39] Ewels PA, Peltzer A, Fillinger S, Patel H, Alneberg J, Wilm A, et al. The nf-core framework for community-curated bioinformatics pipelines. Nat Biotechnol. 2020;38:3. 32055031 10.1038/s41587-020-0439-x

[40] Merkel D. Docker: lightweight Linux containers for consistent development and deployment. Linux J. 2014;2014:239.

[41] Andrews S. FastQC—A quality control tool for high throughput sequence data. Babraham Bioinformatics. 2010. http://www.bioinformatics.babraham.ac.uk/projects/fastqc/. Accessed July 14, 2024.

[42] Martin M. Cutadapt removes adapter sequences from high-throughput sequencing reads. EMBnet J. 2011;17:1.

[43] Seemann T. barrnap 0.9 : rapid ribosomal RNA prediction. GithubCom. 2013. https://github.com/tseemann/barrnap/blob/0.9/README.md. Accessed 28 Apr 2013.

[44] Callahan BJ, McMurdie PJ, Rosen MJ, Han AW, Johnson AJA, Holmes SP. DADA2: high-resolution sample inference from Illumina amplicon data. Nat Methods. 2016;13:7. 27214047 10.1038/nmeth.3869 PMC4927377

[45] Quast C, Pruesse E, Yilmaz P, Gerken J, Schweer T, Yarza P, et al. The SILVA ribosomal RNA gene database project: Improved data processing and web-based tools. Nucleic Acids Res. 2013;41:D1. 23193283 10.1093/nar/gks1219 PMC3531112

[46] Audsley MD, Seleznev A, Joubert DA, Woolfit M, O’Neill SL, McGraw EA. *Wolbachia* infection alters the relative abundance of resident bacteria in adult *Aedes aegypti* mosquitoes, but not larvae. Mol Ecol. 2018;27:297–309. 29165845 10.1111/mec.14436

[47] Schloss PD. Rarefaction is currently the best approach to control for uneven sequencing effort in amplicon sequence analyses. mSphere. 2024;9:2. 10.1128/msphere.00354-23 PMC10900887 38251877

[48] Bolyen E, Rideout JR, Dillon MR, Bokulich NA, Abnet CC, Al-Ghalith GA, et al. Reproducible, interactive, scalable and extensible microbiome data science using QIIME 2. Nat Biotechnol. 2019;37:852–7. 31341288 10.1038/s41587-019-0209-9 PMC7015180

[49] Kers JG, Saccenti E. The power of microbiome studies: some considerations on which alpha and beta metrics to use and how to report results. Front Microbiol. 2022;12:796025. 35310396 10.3389/fmicb.2021.796025 PMC8928147

[50] Lin H, Peddada SD. Multigroup analysis of compositions of microbiomes with covariate adjustments and repeated measures. Nat Methods. 2024;21:1. 38158428 10.1038/s41592-023-02092-7 PMC10776411

[51] Lin H, Eggesbø M, Peddada SD. Linear and nonlinear correlation estimators unveil undescribed taxa interactions in microbiome data. Nat Commun. 2022;13:4946. 35999204 10.1038/s41467-022-32243-x PMC9399263

[52] R Core Team. R Core Team 2021 R: a language and environment for statistical computing. R foundation for statistical computing. R Foundation for Statistical Computing. 2022. https://www.R-project.org/. Accessed July 16, 2024.

[53] Reynolds JA, Poelchau MF, Rahman Z, Armbruster PA, Denlinger DL. Transcript profiling reveals mechanisms for lipid conservation during diapause in the mosquito, *Aedes**albopictus*. J Insect Physiol. 2012;58:7. 22579567 10.1016/j.jinsphys.2012.04.013 PMC3389261

[54] Schmittgen TD, Livak KJ. Analysis of relative gene expression data using real-time quantitative PCR and the 2(-Delta Delta C(T)) method. Methods. 2001;25:4. 11846609 10.1006/meth.2001.1262

[55] Scolari F, Casiraghi M, Bonizzoni M. *Aedes* spp. and their microbiota: a review. Front Microbiol. 2019;10:2036. 31551973 10.3389/fmicb.2019.02036 PMC6738348

[56] Angleró-Rodríguez YI, Tikhe CV, Kang S, Dimopoulos G. *Aedes aegypti* Toll pathway is induced through dsRNA sensing in endosomes. Dev Comp Immunol. 2021;122:104138. 34022257 10.1016/j.dci.2021.104138

[57] Valanne S, Wang JH, Rämet M. The *Drosophila* Toll signaling pathway. J Immunol. 2011;186:2. 10.4049/jimmunol.1002302 21209287

[58] Zhang R, Zhu Y, Pang X, Xiao X, Zhang R, Cheng G. Regulation of antimicrobial peptides in *Aedes aegypti* Aag2 Cells. Front Cell Infect Microbiol. 2017;7:22. 28217557 10.3389/fcimb.2017.00022 PMC5291090

[59] Tang H, Kambris Z, Lemaitre B, Hashimoto C. A serpin that regulates immune melanization in the respiratory system of *Drosophila*. Dev Cell. 2008;15:4. 10.1016/j.devcel.2008.08.017 PMC2671232 18854145

[60] Benoit JB, Vigneron A, Broderick NA, Wu Y, Sun JS, Carlson JR, et al. Symbiont-induced odorant binding proteins mediate insect host hematopoiesis. Elife. 2017;6:e19535. 28079523 10.7554/eLife.19535 PMC5231409

[61] Halbach R, Miesen P, van Rij RP. Zooming in on targets of mosquito small RNAs. Trends Parasitol. 2021;37:687–9. 34147336 10.1016/j.pt.2021.06.002

[62] Nappi AJ, Christensen B. Melanogenesis and associated cytotoxic reactions: applications to insect innate immunity. Insect Biochem Mol Biol. 2005;35:443–59. 15804578 10.1016/j.ibmb.2005.01.014

[63] Crean AJ, Monro K, Marshall DJ. Fitness consequences of larval traits persist across the metamorphic boundary. Evolution (NY). 2011;65:11. 10.1111/j.1558-5646.2011.01372.x 22023576

[64] Moltini-Conclois I, Stalinski R, Tetreau G, Després L, Lambrechts L. Larval exposure to the bacterial insecticide Bti enhances dengue virus susceptibility of adult *Aedes aegypti* mosquitoes. Insects. 2018;9:4. 30558130 10.3390/insects9040193 PMC6316598

[65] Westbrook CJ, Reiskind MH, Pesko KN, Greene KE, Lounibos LP. Larval environmental temperature and the susceptibility of *Aedes albopictus* skuse (Diptera: Culicidae) to chikungunya virus. Vector Borne Zoonotic Dis. 2010;10:3. 19725768 10.1089/vbz.2009.0035 PMC2883477

[66] Telang A, Qayum AA, Parker A, Sacchetta BR, Byrnes GR. Larval nutritional stress affects vector immune traits in adult yellow fever mosquito *Aedes aegypti (Stegomyia aegypti)*. Med Vet Entomol. 2012;26:3. 10.1111/j.1365-2915.2011.00993.x 22112201

[67] Dickson LB, Jiolle D, Minard G, Moltini-Conclois I, Volant S, Ghozlane A, et al. Carryover effects of larval exposure to different environmental bacteria drive adult trait variation in a mosquito vector. Sci Adv. 2017;3:8. 10.1126/sciadv.1700585 PMC5559213 28835919

[68] Flores AE, Garcia GP, Badii MH, Tovar MLR, Salas IF. Effects of sublethal concentrations of Vectobac^®^ on biological parameters of *Aedes aegypti*. J Am Mosq Control Assoc. 2004;20:4. 15669383

[69] Wang LY, Jaal Z. Sublethal effects of *Bacillus thuringiensis* H-14 on the survival rate, longevity, fecundity and F1 generation developmental period of *Aedes aegypti*. Dengue Bull. 2005;29:192–6.

[70] Simsek FM, Akiner MM, Caglar SS. Effects of sublethal concentrations of vectobac 12 as on some biological parameters of the malaria vector *Anopheles superpictus*. J Anim Vet Adv. 2009;8:7.

[71] Coon KL, Brown MR, Strand MR. Mosquitoes host communities of bacteria that are essential for development but vary greatly between local habitats. Mol Ecol. 2016;25:22. 10.1111/mec.13877 PMC5118126 27718295

[72] Moll RM, Romoser WS, Modrzakowski MC, Moncayo AC, Lerdthusnee K. Meconial peritrophic membranes and the fate of midgut bacteria during mosquito (Diptera: Culicidae) metamorphosis. J Med Entomol. 2001;38:1. 11268687 10.1603/0022-2585-38.1.29

[73] Mosquera KD, Martinez Villegas LE, Pidot SJ, Sharif C, Klimpel S, Stinear TP, et al. Multi-Omic analysis of symbiotic bacteria associated with *Aedes aegypti* breeding sites. Front Microbiol. 2021;12:703711. 34475861 10.3389/fmicb.2021.703711 PMC8406634

[74] Harrison RE, Yang X, Eum JH, Martinson VG, Dou X, Valzania L, et al. The mosquito *Aedes aegypti* requires a gut microbiota for normal fecundity, longevity and vector competence. Commun Biol. 2023;6:1154. 37957247 10.1038/s42003-023-05545-z PMC10643675

[75] Taracena-Agarwal ML, Walter-Nuno AB, Bottino-Rojas V, Mejia APG, Xu K, Segal S, et al. Juvenile Hormone as a contributing factor in establishing midgut microbiota for fecundity and fitness enhancement in adult female *Aedes aegypti*. Commun Biol. 2024;7:687. 38839829 10.1038/s42003-024-06334-y PMC11153597

[76] Caragata EP, Tikhe CV, Dimopoulos G. Curious entanglements: interactions between mosquitoes, their microbiota, and arboviruses. Curr Opin Virol. 2019;37:26–36. 31176069 10.1016/j.coviro.2019.05.005 PMC6768729

[77] Eren AM, Sogin ML, Morrison HG, Vineis JH, Fisher JC, Newton RJ, et al. A single genus in the gut microbiome reflects host preference and specificity. ISME J. 2015;9:1. 24936765 10.1038/ismej.2014.97 PMC4274434

[78] Minard G, Tran FH, Dubost A, Tran-Van V, Mavingui P, Valiente MC. Pyrosequencing 16S rRNA genes of bacteria associated with wild tiger mosquito *Aedes albopictus*: a pilot study. Front Cell Infect Microbiol. 2014;4:59. 24860790 10.3389/fcimb.2014.00059 PMC4030203

[79] Pinos D, Andrés-Garrido A, Ferré J, Hernández-Martínez P. Response mechanisms of invertebrates to *Bacillus thuringiensis* and its pesticidal proteins. Microbiol Mol Biol Rev. 2021;85:10–1128. 10.1128/MMBR.00007-20 PMC8549848 33504654

[80] Despres L, Stalinski R, Faucon F, Navratil V, Viari A, Paris M. Chemical and biological insecticides select distinct gene expression patterns in *Aedes aegypti* mosquito. Biol Lett. 2014;10:20140716. 25540155 10.1098/rsbl.2014.0716 PMC4298186

[81] Stalinski R, Laporte F, Després L, Tetreau G. Alkaline phosphatases are involved in the response of *Aedes**aegypti* larvae to intoxication with *Bacillus**thuringiensis* subsp. *israelensis* Cry toxins. Environ Microbiol. 2016;18:3. 10.1111/1462-2920.13186 26663676

[82] Moore AJ, Beazley WD, Bibby MC, Devine DA. Antimicrobial activity of cecropins. J Antimicrob Chemother. 1996;37:6. 10.1093/jac/37.6.1077 8836811

[83] Strand MR. Composition and functional roles of the gut microbiota in mosquitoes. Curr Opin Insect Sci. 2018;28:59–65. 30551768 10.1016/j.cois.2018.05.008 PMC6296257

[84] Macdonald G. Epidemiologic models in studies of vector-borne diseases: the re dyer lecture. Public Health Rep. 1961;76:9. PMC1929763 13764730

[85] Finney DJ, Tattersfield F. Probit analysis: a statistical treatment of the sigmoid response curve. J Roy Stat Soc. 1947;110:263.

