# Supplementary figures and images for: Carry-over effects of Bacillus thuringiensis on tolerant Aedes albopictus mosquitoes
Source: Parasit Vectors. 2024 Nov 7;17:456. doi: 10.1186/s13071-024-06556-3 (PMC11545555; doi:10.1186/s13071-024-06556-3)

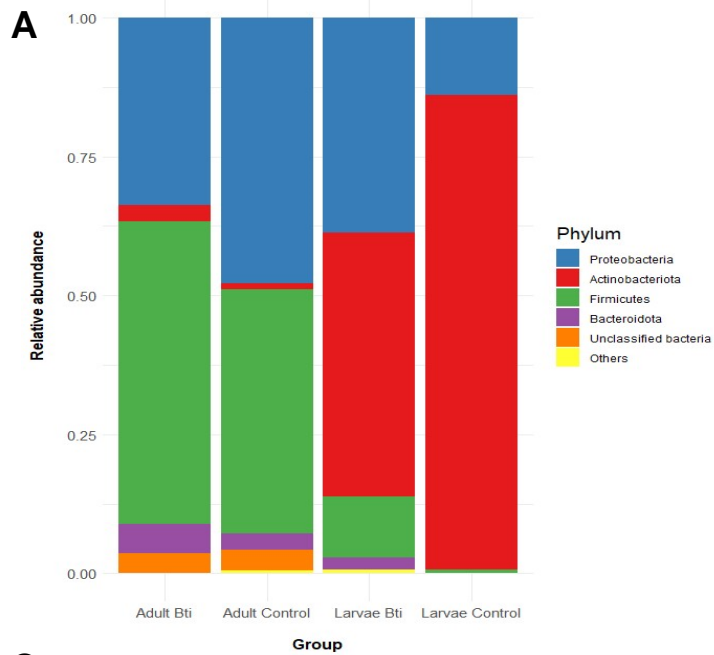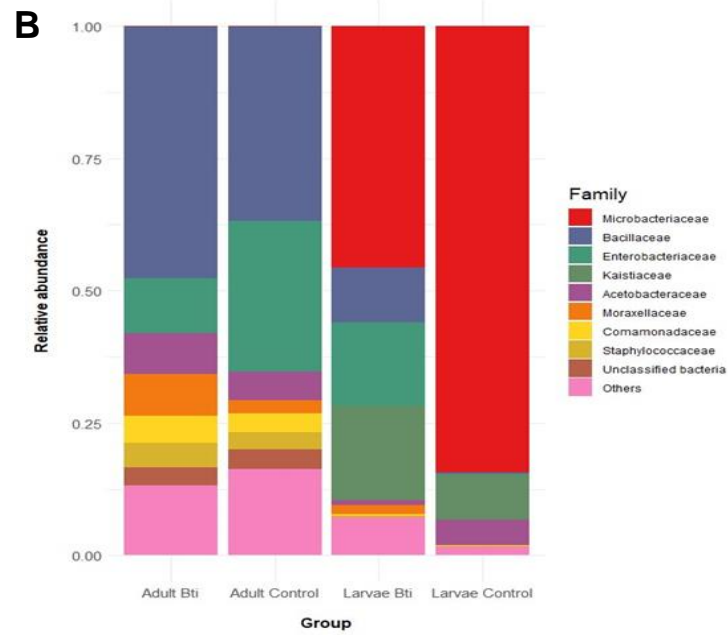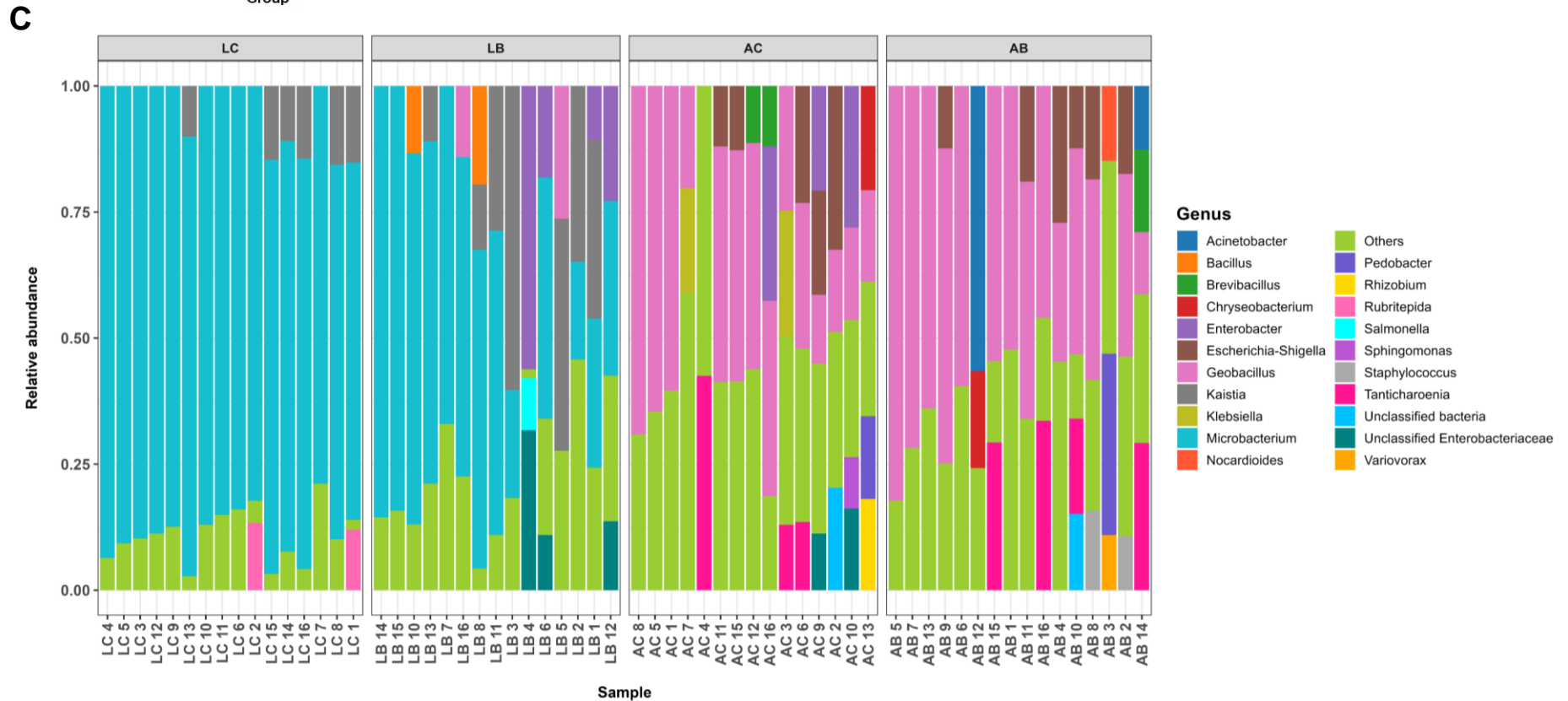

Supplement: Supplementary file 1 — Additional file 1: Figure S1. Rarefaction analyses to ensure the unbiased comparisons of species richness and diversity metrics across all samples. The rarefaction depth of 4000 sequences was chosen on the basis of the rarefaction curves. Samples with a rarefaction depth lower than this threshold were discarded from diversity analysis. [file 13071_2024_6556_MOESM1_ESM.pdf]

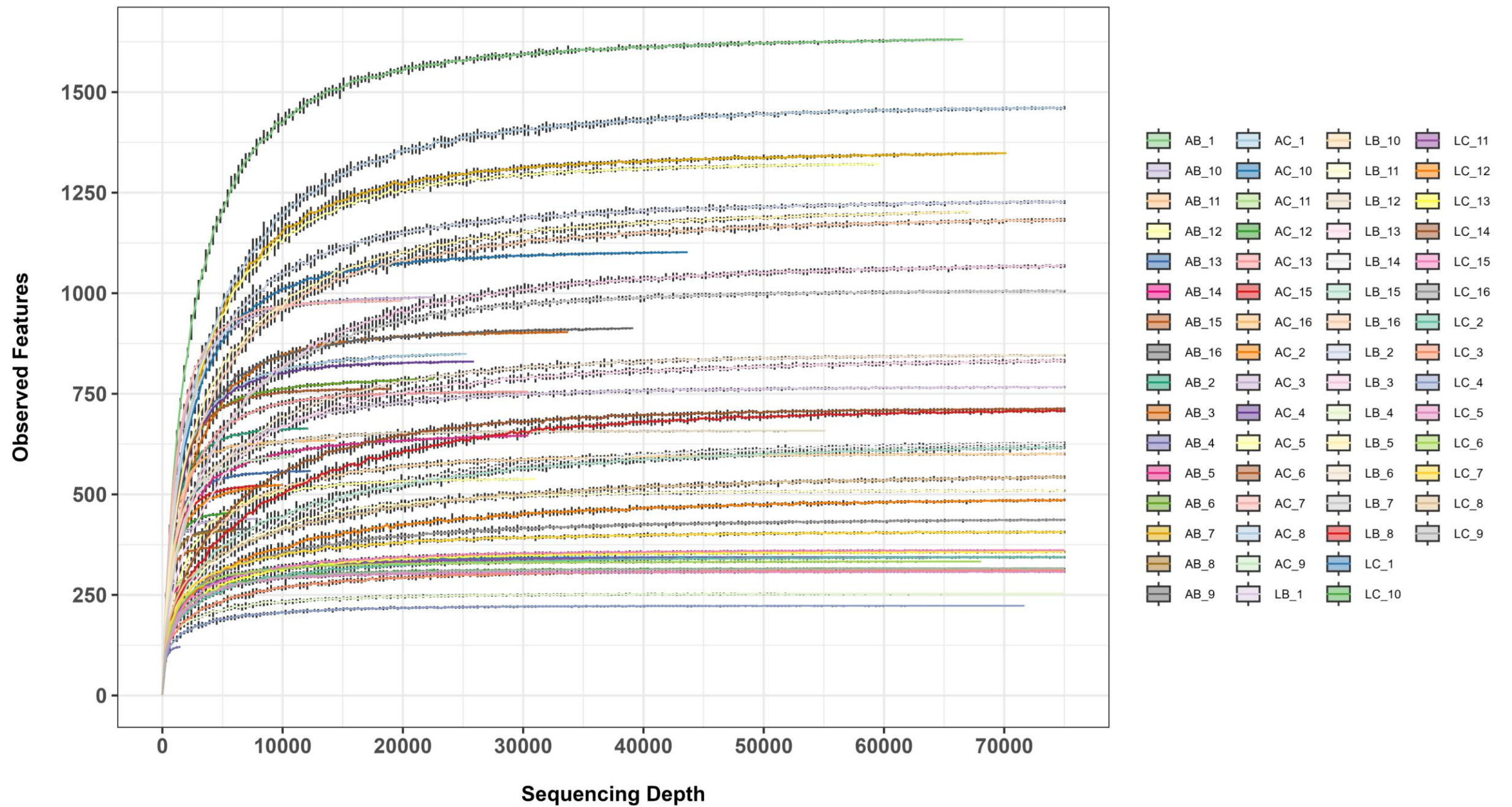

Supplement: Supplementary file 4 — Additional file 4: Table S2. Comparison of values of life-table parameters in tolerant and control larvae, emerging adults, and the progeny from these adults (F1). [file 13071_2024_6556_MOESM4_ESM.pdf]

A

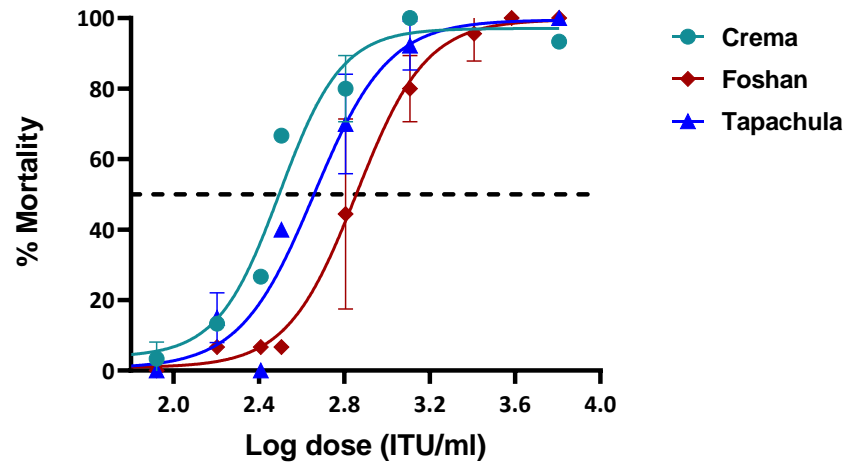

B

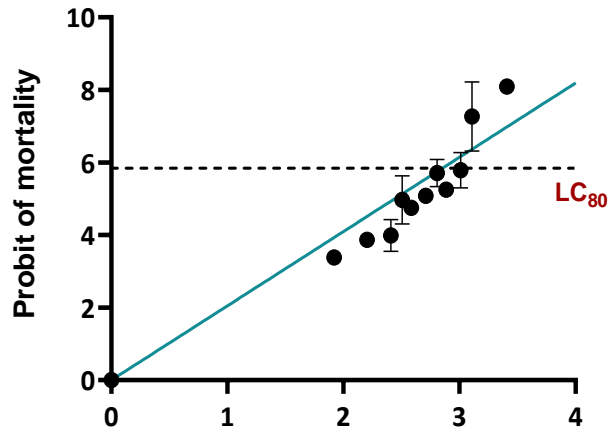

Supplement: Supplementary file 6 — Additional file 6: Table S3. Summary of number of sequences with DADA2 filtering. [file 13071_2024_6556_MOESM6_ESM.pdf]

**A**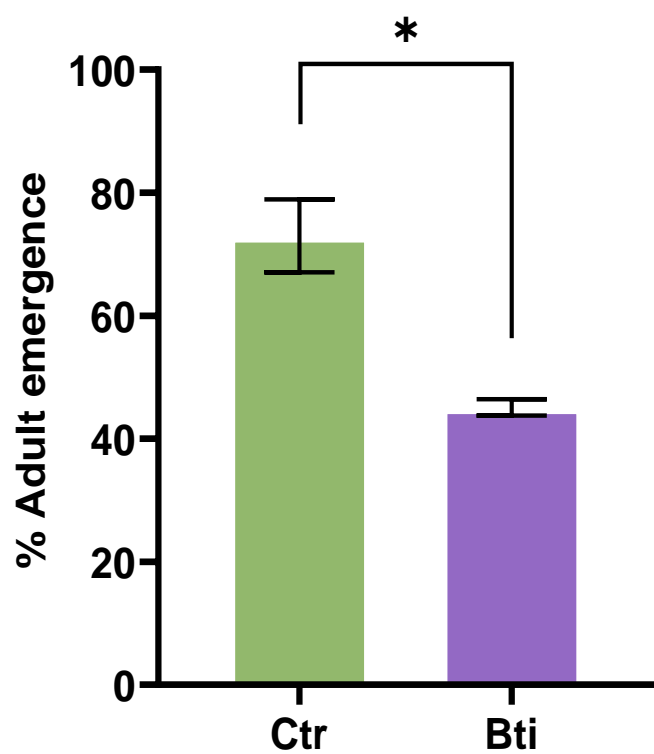**B**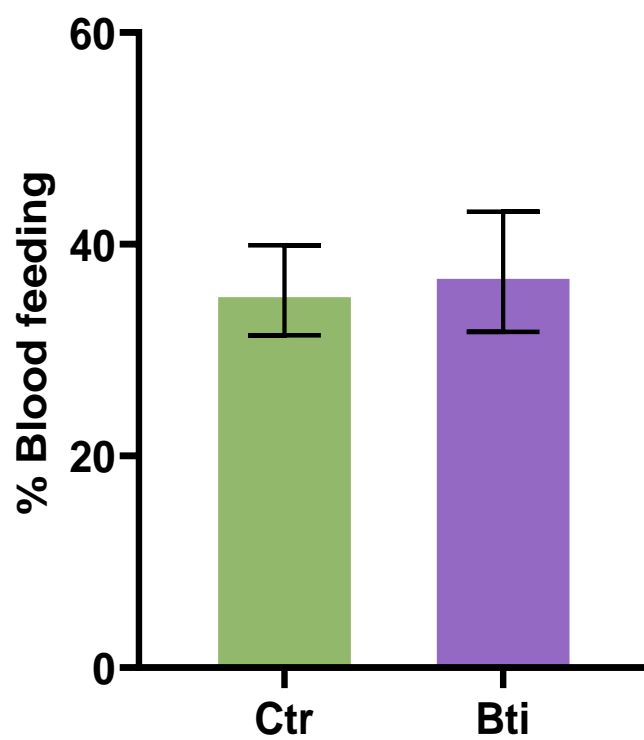**C**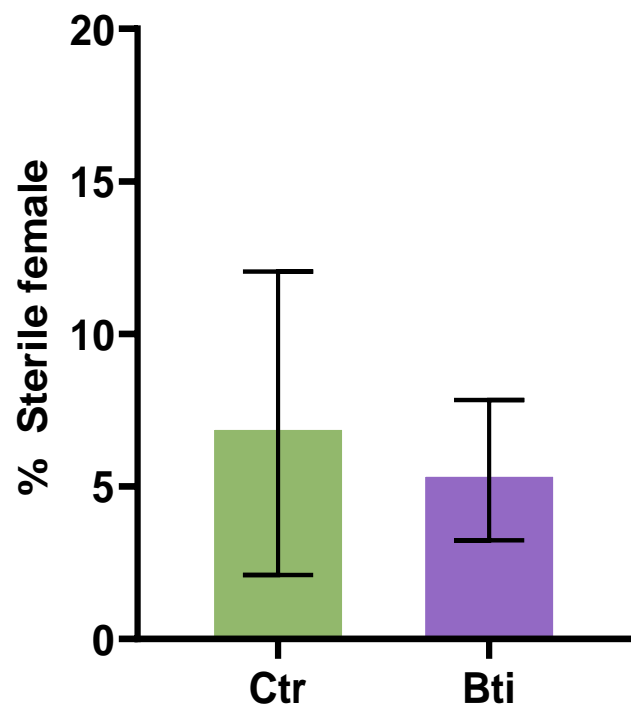**D**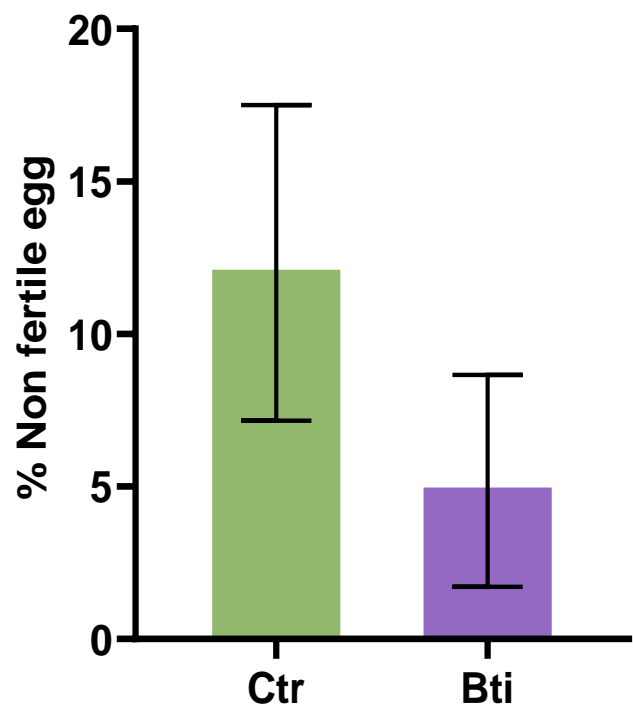

Supplement: Supplementary file 8 — Additional file 8: Table S5. Top 50 most abundant genera based on relative abundance. [file 13071_2024_6556_MOESM8_ESM.pdf]
