# Supplementary material for: Carry-over effects of Bacillus thuringiensis on tolerant Aedes albopictus mosquitoes
Source: Parasit Vectors. 2024 Nov 7;17:456. doi: 10.1186/s13071-024-06556-3 (PMC11545555; doi:10.1186/s13071-024-06556-3)
Supplement: Supplementary file 2 — Additional file 2: Table S1. List of primers for gene expression analysis. [file 13071_2024_6556_MOESM2_ESM.pdf]

Additional File 11: Table S7. Common bacterial genera across groups

						Larvae Control		Larvae Bti		Adult Control		Adult Bti	
**Kingdom**	**Phylum**	**Class**	**Order**	**Family**	**Genus**	**Mean_Abundance_LC**	**SD_Abundance_LC**	**Mean_Abundance_LB**	**SD_Abundance_LB**	**Mean_Abundance_AC**	**SD_Abundance_AC**	**Mean_Abundance_AB**	**SD_Abundance_AB**
Bacteria	Actinobacteriota	Actinobacteria	Corynebacteriales	Corynebacteriaceae	Corynebacterium	0.000038	0.000116	0.000085	0.000164	0.000416	0.000542	0.001081	0.001687
Bacteria	Actinobacteriota	Actinobacteria	Corynebacteriales	Corynebacteriaceae	Lawsonella	0.000050	0.000064	0.000234	0.000373	0.000254	0.000431	0.000949	0.001926
Bacteria	Actinobacteriota	Actinobacteria	Corynebacteriales	Mycobacteriaceae	Mycobacterium	0.000039	0.000087	0.000075	0.000143	0.000031	0.000121	0.000032	0.000087
Bacteria	Actinobacteriota	Actinobacteria	Corynebacteriales	Nocardiaceae	Rhodococcus	0.000105	0.000199	0.000025	0.000068	0.000500	0.000768	0.000888	0.001441
Bacteria	Actinobacteriota	Actinobacteria	Micrococcales	Microbacteriaceae	Microbacterium	0.825708	0.067494	0.482734	0.274665	0.000471	0.000920	0.000683	0.001654
Bacteria	Actinobacteriota	Actinobacteria	Propionibacteriales	Propionibacteriaceae	Cutibacterium	0.000102	0.000072	0.000251	0.000301	0.000770	0.000906	0.001064	0.001354
Bacteria	Bacteroidota	Bacteroidia	Cytophagales	Cytophagaceae	Siphonobacter	0.000007	0.000020	0.000553	0.000973	0.000453	0.001303	0.000039	0.000135
Bacteria	Bacteroidota	Bacteroidia	Flavobacteriales	Weeksellaceae	Chryseobacterium	0.000178	0.000285	0.003896	0.009169	0.005201	0.018650	0.004219	0.011613
Bacteria	Bacteroidota	Bacteroidia	Sphingobacteriales	Sphingobacteriaceae	Pedobacter	0.000019	0.000074	0.000015	0.000058	0.003987	0.014837	0.009485	0.033907
Bacteria	Firmicutes	Bacilli	Bacillales	Bacillaceae	Bacillus	0.000006	0.000025	0.046464	0.056897	0.000231	0.000406	0.000202	0.000271
Bacteria	Firmicutes	Bacilli	Bacillales	Bacillaceae	Geobacillus	0.004190	0.006810	0.040352	0.072110	0.099780	0.151173	0.109201	0.161246
Bacteria	Firmicutes	Bacilli	Brevibacillales	Brevibacillaceae	Brevibacillus	0.000702	0.001627	0.001587	0.002822	0.006863	0.010175	0.002035	0.004045
Bacteria	Firmicutes	Bacilli	Lactobacillales	Lactobacillaceae	Lactobacillus	0.000216	0.000245	0.000041	0.000161	0.000400	0.000525	0.000356	0.000689
Bacteria	Firmicutes	Bacilli	Lactobacillales	Streptococcaceae	Streptococcus	0.000009	0.000038	0.000136	0.000264	0.000562	0.001069	0.000883	0.001621
Bacteria	Firmicutes	Bacilli	Staphylococcales	Staphylococcaceae	Staphylococcus	0.000992	0.000874	0.001766	0.001588	0.007464	0.009352	0.008636	0.011133
Bacteria	Firmicutes	Clostridia	Peptostreptococcales-Tissierellales	Family XI	Finegoldia	0.000005	0.000021	0.000010	0.000038	0.000079	0.000211	0.000155	0.000502
Bacteria	Proteobacteria	Alphaproteobacteria	Caulobacterales	Caulobacteraceae	Brevundimonas	0.000055	0.000155	0.000012	0.000047	0.000409	0.000568	0.000110	0.000272
Bacteria	Proteobacteria	Alphaproteobacteria	Rhizobiales	Beijerinckiaceae	Bosea	0.000132	0.000204	0.004613	0.009747	0.000413	0.001042	0.000029	0.000116
Bacteria	Proteobacteria	Alphaproteobacteria	Rhizobiales	Beijerinckiaceae	Methylobacterium-Methylorubrum	0.001359	0.002125	0.005771	0.007083	0.001493	0.002249	0.001655	0.002694
Bacteria	Proteobacteria	Alphaproteobacteria	Rhizobiales	Rhizobiaceae	Allorhizobium-Neorhizobium-Pararhizobium-Rhizobium	0.000501	0.000694	0.005269	0.015155	0.006955	0.016194	0.000201	0.000412
Bacteria	Proteobacteria	Alphaproteobacteria	Rhodobacterales	Rhodobacteraceae	Paracoccus	0.000005	0.000018	0.000033	0.000127	0.000286	0.000332	0.000257	0.000601
Bacteria	Proteobacteria	Alphaproteobacteria	Rickettsiales	Anaplasmataceae	Wolbachia	0.017843	0.029613	0.005744	0.009775	0.755992	0.214488	0.787938	0.243137
Bacteria	Proteobacteria	Alphaproteobacteria	Sphingomonadales	Sphingomonadaceae	Sphingomonas	0.000145	0.000261	0.000450	0.000519	0.005771	0.009469	0.001329	0.002317
Bacteria	Proteobacteria	Gammaproteobacteria	Burkholderiales	Comamonadaceae	Aquabacterium	0.001154	0.001830	0.000907	0.000900	0.003709	0.003082	0.005243	0.008570

Bacteria	Proteobacteria	Gammaproteobacteria	Burkholderiales	Comamonadaceae	Delftia	0.000013	0.000053	0.001242	0.002832	0.000692	0.002272	0.000849	0.001862
Bacteria	Proteobacteria	Gammaproteobacteria	Burkholderiales	Comamonadaceae	Leptothrix	0.000009	0.000038	0.000008	0.000032	0.000212	0.000279	0.000446	0.000904
Bacteria	Proteobacteria	Gammaproteobacteria	Burkholderiales	Oxalobacteraceae	Massilia	0.000089	0.000104	0.000214	0.000268	0.000584	0.000635	0.000765	0.001171
Bacteria	Proteobacteria	Gammaproteobacteria	Enterobacterales	Enterobacteriaceae	Enterobacter	0.000011	0.000044	0.080329	0.142676	0.014773	0.026053	0.000076	0.000212
Bacteria	Proteobacteria	Gammaproteobacteria	Enterobacterales	Enterobacteriaceae	Escherichia-Shigella	0.001509	0.000933	0.002610	0.002235	0.017569	0.016204	0.009472	0.009847
Bacteria	Proteobacteria	Gammaproteobacteria	Pseudomonadales	Moraxellaceae	Acinetobacter	0.000014	0.000034	0.006187	0.010440	0.007339	0.011609	0.018208	0.033039
Bacteria	Proteobacteria	Gammaproteobacteria	Pseudomonadales	Moraxellaceae	Enhydrobacter	0.000159	0.000334	0.000120	0.000220	0.000700	0.001261	0.000364	0.000936
Bacteria	Proteobacteria	Gammaproteobacteria	Pseudomonadales	Pseudomonadaceae	Pseudomonas	0.000029	0.000063	0.000617	0.001400	0.002802	0.005342	0.001408	0.003336
Bacteria	Proteobacteria	Gammaproteobacteria	Salinisphaerales	Solimonadaceae	Hydrocarboniphaga	0.000006	0.000022	0.000005	0.000020	0.000052	0.000125	0.000220	0.000777
