# Supplementary material for: Carry-over effects of Bacillus thuringiensis on tolerant Aedes albopictus mosquitoes
Source: Parasit Vectors. 2024 Nov 7;17:456. doi: 10.1186/s13071-024-06556-3 (PMC11545555; doi:10.1186/s13071-024-06556-3)
Supplement: Supplementary file 3 — Additional file 3: Figure S2. Susceptibility of three different laboratory populations of Aedes albopictus to Bti-based bio-insecticide. A Dose–response curve of Foshan, Tapachula, and Crema populations exposed to different concentrations of Bti. Dashed line refers to the log dose of the LC50. B Probit analysis of Crema strain. The plot illustrates the transformation of the dose–response curve into the linear regression between the log dose and probit of mortality on the basis of the Finney’s table [85]. The dashed line refers to the log dose of the LC80. [file 13071_2024_6556_MOESM3_ESM.pdf]

Additional File 12: Table S8. Statistical analysis of differentially abundant genus in LB vs AB (A), AB vs AC (B) and LB vs LC (C).

A) **LB vs LC**

**Kingdom**	**Phylum**	**Class**	**Order**	**Family**	**Genus**	**lfc**	**se**	**W**	**p_value**	**q_value**
Bacteria	Proteobacteria	Gammaproteobacteria	Pseudomonadales	Moraxellaceae	Acinetobacter	3.206	0.646	4.962	0.000	0.000
Bacteria	Proteobacteria	Gammaproteobacteria	Enterobacterales	Enterobacteriaceae	Enterobacter	4.322	0.755	5.722	0.000	0.000
Bacteria	Proteobacteria	Gammaproteobacteria	Burkholderiales	Comamonadaceae	Delftia	2.661	0.615	4.326	0.002	0.012
Bacteria	Firmicutes	Bacilli	Bacillales	Bacillaceae	Bacillus	6.112	0.694	8.802	0.000	0.000
Bacteria	Proteobacteria	Alphaproteobacteria	Rhizobiales	Beijerinckiaceae	Bosea	2.484	0.663	3.747	0.002	0.011
Bacteria	Bacteroidota	Bacteroidia	Cytophagales	Cytophagaceae	Siphonobacter	2.746	0.584	4.699	0.001	0.007

B) **AB vs AC**

**Kingdom**	**Phylum**	**Class**	**Order**	**Family**	**Genus**	**lfc**	**se**	**W**	**p_value**	**q_value**
Bacteria	Proteobacteria	Alphaproteobacteria	Rhizobiales	Rhizobiaceae	Allorhizobium-Neorhizobium-Pararhizobium-Rhizobium	-2.025	0.593	-3.413	0.002	0.011
Bacteria	Proteobacteria	Gammaproteobacteria	Enterobacterales	Enterobacteriaceae	Enterobacter	-2.519	0.677	-3.723	0.001	0.003

C) **LB vs AB**

**Kingdom**	**Phylum**	**Class**	**Order**	**Family**	**Genus**	**lfc**	**se**	**W**	**p_value**	**q_value**
Bacteria	Unknown	Unknown	Unknown	Unknown	Unknown	-2.153	0.544	-3.955	0.000	0.001
Bacteria	Proteobacteria	Alphaproteobacteria	Rhizobiales	Rhizobiaceae	Allorhizobium-Neorhizobium-Pararhizobium-Rhizobium	2.388	0.625	3.821	0.001	0.004
Bacteria	Firmicutes	Bacilli	Bacillales	Bacillaceae	Geobacillus	-1.887	0.702	-2.686	0.009	0.045
Bacteria	Actinobacteriota	Actinobacteria	Micrococcales	Microbacteriaceae	Microbacterium	6.926	0.651	10.632	0.000	0.000

Bacteria	Proteobacteria	Gammaproteobacteria	Enterobacterales	Enterobacteriaceae	Escherichia-Shigella	-1.650	0.523	-3.155	0.003	0.010
Bacteria	Proteobacteria	Gammaproteobacteria	Enterobacterales	Enterobacteriaceae	Enterobacter	3.746	0.807	4.641	0.000	0.000
Bacteria	Actinobacteriota	Actinobacteria	Corynebacteriales	Corynebacteriaceae	Corynebacterium	-1.820	0.603	-3.018	0.005	0.037
Bacteria	Proteobacteria	Alphaproteobacteria	Rickettsiales	Anaplasmataceae	Wolbachia	-6.216	0.694	-8.962	0.000	0.000
Bacteria	Firmicutes	Bacilli	Bacillales	Bacillaceae	Bacillus	4.223	0.757	5.575	0.000	0.000
Bacteria	Proteobacteria	Alphaproteobacteria	Rhizobiales	Beijerinckiaceae	Bosea	2.847	0.711	4.004	0.001	0.006

D) LC vs AC

Kingdom	Phylum	Class	Order	Family	Genus	lfc	se	W	p_value	q_value
Bacteria	Firmicutes	Bacilli	Staphylococcales	Staphylococcaceae	Staphylococcus	-1.891	0.444	-4.256	0.000	0.001
Bacteria	Unknown	Unknown	Unknown	Unknown	Unknown	-3.170	0.505	-6.281	0.000	0.000
Bacteria	Proteobacteria	Gammaproteobacteria	Pseudomonadales	Moraxellaceae	Acinetobacter	-3.896	0.607	-6.413	0.000	0.000
Bacteria	Firmicutes	Bacilli	Bacillales	Bacillaceae	Geobacillus	-3.118	0.503	-6.202	0.000	0.000
Bacteria	Firmicutes	Bacilli	Brevibacillales	Brevibacillaceae	Brevibacillus	-2.050	0.659	-3.112	0.003	0.023
Bacteria	Proteobacteria	Gammaproteobacteria	Burkholderiales	Comamonadaceae	Aquabacterium	-1.380	0.463	-2.980	0.004	0.025
Bacteria	Actinobacteriota	Actinobacteria	Micrococcales	Microbacteriaceae	Microbacterium	7.976	0.544	14.653	0.000	0.000
Bacteria	Proteobacteria	Gammaproteobacteria	Enterobacterales	Enterobacteriaceae	Escherichia-Shigella	-2.286	0.461	-4.958	0.000	0.000
Bacteria	Proteobacteria	Gammaproteobacteria	Enterobacterales	Enterobacteriaceae	Enterobacter	-3.246	0.682	-4.760	0.000	0.000
Bacteria	Actinobacteriota	Actinobacteria	Propionibacteriales	Propionibacteriaceae	Cutibacterium	-1.527	0.515	-2.966	0.005	0.027
Bacteria	Proteobacteria	Gammaproteobacteria	Pseudomonadales	Pseudomonadaceae	Pseudomonas	-2.212	0.593	-3.733	0.001	0.006
Bacteria	Proteobacteria	Alphaproteobacteria	Rickettsiales	Anaplasmataceae	Wolbachia	-5.187	0.709	-7.314	0.000	0.000

Bacteria	Proteobacteria	Alphaproteobacteria	Sphingomonadales	Sphingomonadaceae	Sphingomonas	-2.567	0.563	-4.558	0.000	0.000
Bacteria	Firmicutes	Bacilli	Bacillales	Bacillaceae	Bacillus	-2.858	0.560	-5.105	0.000	0.000
Bacteria	Bacteroidota	Bacteroidia	Cytophagales	Cytophagaceae	Siphonobacter	-3.166	0.616	-5.135	0.001	0.004

Only the genera that are differentially abundant (q< 0.05) and passed sensitivity analysis (passed_ss = TRUE) are reported.
