# Supplementary material for: Carry-over effects of Bacillus thuringiensis on tolerant Aedes albopictus mosquitoes
Source: Parasit Vectors. 2024 Nov 7;17:456. doi: 10.1186/s13071-024-06556-3 (PMC11545555; doi:10.1186/s13071-024-06556-3)
Supplement: Supplementary file 5 — Additional file 5: Figure S3. Fitness comparisons between Bti-tolerant and control adults. Percentage of adult emergence (A), blood feeding rate (B), sterile female (C), and non-fertile eggs (D) of Bti and control groups. Green and purple colors represent control samples and Bti-tolerant samples, respectively. Comparison of the fitness parameters between Bti-tolerant and control samples was performed using Student’s t-test. *Represents P-value < 0.05. [file 13071_2024_6556_MOESM5_ESM.pdf]

Additional file 2: Table S1. List of primers for gene expression analysis.

Target gene	VectorBase/Gene Bank		Seq 5' to 3'	Amplicon size (bp)
Myd 88	AALC636_030222	FOR	AGTTCGAACAGATTCCCCAGT	132
		REV	TTGTTGACTTTTGTGCCGCC	
Cactous	AALFPA_063808	FOR	AGTCCGGAAACCAGAAGCAG	140
		REV	GTCTTTGCACAGGACCCTCA	
Cecropin B	AALFPA_043934	FOR	GCGCTTGTTCTGCTTATCGG	142
		REV	ATCCGGTGAGTACGGGAAGA	
Cecropin A	AALF012131	FOR	CCGCAGTCGTAAGGATCCAA	137
		REV	TTGACCCAGCAGGACCAAAG	
Drosal	AALFPA_074733	FOR	ACAACTGAAGAGGCCATCGG	103
		REV	GTCACGCTTCAGTGACCAGA	
Dicer 2	AALFPA_062753	FOR	CGTTACCATGGTGCATGAGC	129
		REV	TCGATGAGGAATTGATCCGGC	
Ago 2	AALFPA_066143	FOR	GGCTCCGACCTACTATGCAC	102
		REV	ATCCTCTCGTACTCGTTCGC	
PPO	AALFPA_056936	FOR	TCGATCTTCCGGACAATTACCT	97
		REV	ACGGGAATTCTGGTTTGCAC	
OBP 28	AALFPA_044806	FOR	TGAAATCCTCAACTGCGCCT	122
		REV	ATCCGGGTGACTTCATCAGC	
RpL34	AALC636_026691	FOR	AGAAGCTCAGCGGAATCAAGCCATCGCG	263
		REV	GGGCTCGTCTACCACGTTTACTTGCTCTTGC	
