# Supplementary material for: Carry-over effects of Bacillus thuringiensis on tolerant Aedes albopictus mosquitoes
Source: Parasit Vectors. 2024 Nov 7;17:456. doi: 10.1186/s13071-024-06556-3 (PMC11545555; doi:10.1186/s13071-024-06556-3)
Supplement: Supplementary file 7 — Additional file 7: Table S4. Average relative abundance of sequences assigned to a given bacterial taxon at genus level. [file 13071_2024_6556_MOESM7_ESM.pdf]

Additional file 4: Table S2. Comparison of values of life-table parameters in tolerant and control larvae, emerging adults and the progeny from these adults (F1)

**Comparison of life table parameters**			
**Trait**	**control larvae**	**Bti-tolerant larvae**	**p-value**
Adult emergence	73±5.94	45±1.34	0.0153
	**adults from control larvae**	**adults from Bti-tolerant larvae**	
Female longevity *	24±1.2	10±0.6	≤0.0001
Male longevity *	20±0.96	9±0.47	≤0.0001
Blood feeding rate	35.65±4.28	37.39±5.68	0.838
Percentage of sterile females	7.07±4.97	5.538±2.3	0.302
Fecundity	74.33±4.01	54.0±2.77	0.019
Hatching rate	68.61±11.7	57.46±12.41	0.309
Fertility	53.54±4.34	61.32±3.28	0.223
percentage of non viable eggs	12.33±5.173	5.185±3.47	0.783
Number of larvae per female	39.42±3.61	33.76±2.51	0.188
	**F1 progeny from control larvae**	**F1 progeny from Bti-tolerant larvae**	
Larval developmental time	6.39±3.42	6.98±1.51	0.989
Pupation rate	70.10±8.98	81.12±12.33	0.179
Pupa developmental time	2.95±1.82	2.62±0.76	0.999
sex ratio	42.74±6.31	44.51±8.05	0.589
Eggs to adult viability	33.92±2.90	42.59±11.48	0.309
Male developmental speed	9.88±2.71	10.08±1.87	0.999
Female developmental speed	8.77±3.96	10.08±1.66	0.999
