# Supplementary material for: Carry-over effects of Bacillus thuringiensis on tolerant Aedes albopictus mosquitoes
Source: Parasit Vectors. 2024 Nov 7;17:456. doi: 10.1186/s13071-024-06556-3 (PMC11545555; doi:10.1186/s13071-024-06556-3)
Supplement: Supplementary file 9 — Additional file 9: Table S6. Comparison of diversity metrics of the gut microbiota. [file 13071_2024_6556_MOESM9_ESM.pdf]

Additional file 6: Table S3. Summary of number of sequences with DADA2 filtering

Sample name	DADA2_input	filtered	denoisedF	denoisedR	merged	nonchim	input_tax_filter	filtered_tax_filter	lost	retained_percent	lost_percent
Larvae_Control_1	121248	105665	105136	105292	103922	83288	83288	79875	3413	95.90	4.10
Larvae_Control_2	153008	132500	132071	132252	131568	117470	117470	115331	2139	98.18	1.82
Larvae_Control_3	136765	116730	116505	116611	116058	106477	106477	104985	1492	98.60	1.40
Larvae_Control_4	97535	78880	78690	78753	78478	71928	71928	71911	17	99.98	0.02
Larvae_Control_5	141845	122176	121507	121792	120790	106232	106232	105456	776	99.27	0.73
Larvae_Control_6	103751	87378	86975	87176	86287	77288	77288	68158	9130	88.19	11.81
Larvae_Control_7	145624	123136	122582	122654	121589	105513	105513	104703	810	99.23	0.77
Larvae_Control_8	150284	128202	127482	127712	126454	105167	105167	104500	667	99.37	0.63
Larvae_Control_9	144152	124005	123608	123721	122856	109003	109003	108913	90	99.92	0.08
Larvae_Control_10	120818	104145	103678	103972	103157	90927	90927	87357	3570	96.07	3.93
Larvae_Control_11	141765	117232	116815	116958	116195	105860	105860	105783	77	99.93	0.07
Larvae_Control_12	121737	100887	100479	100633	99813	89738	89738	88323	1415	98.42	1.58
Larvae_Control_13	113397	94059	93780	93866	93272	84231	84231	84142	89	99.89	0.11
Larvae_Control_14	131250	111934	111261	111633	110401	99608	99608	96810	2798	97.19	2.81
Larvae_Control_15	114461	96745	96376	96467	95796	86003	86003	85966	37	99.96	0.04
Larvae_Control_16	111522	94710	94414	94451	93795	85359	85359	85280	79	99.91	0.09
Larvae_BTI_1	133345	111436	110644	110986	108686	84158	84158	81947	2211	97.37	2.63
Larvae_BTI_2	136987	117459	116864	117080	115716	93795	93795	93425	370	99.61	0.39
Larvae_BTI_3	143251	125898	125173	125532	123758	102883	102883	101373	1510	98.53	1.47
Larvae_BTI_4	129573	107129	106835	106900	106291	84107	84107	83969	138	99.84	0.16

Larvae_BTI_5	132014	107741	106688	107179	105109	67965	67965	67456	509	99.25	0.75
Larvae_BTI_6	118470	94525	94004	94171	93086	77982	77982	77749	233	99.70	0.30
Larvae_BTI_7	134444	114849	114484	114597	113742	102213	102213	102038	175	99.83	0.17
Larvae_BTI_8	136490	117528	117018	117292	116367	99743	99743	99630	113	99.89	0.11
Larvae_BTI_10	144530	123233	122766	122969	122216	106825	106825	106594	231	99.78	0.22
Larvae_BTI_11	135974	112743	112069	112388	110939	90130	90130	89907	223	99.75	0.25
Larvae_BTI_12	94754	70744	70044	70402	68986	55433	55433	55142	291	99.48	0.52
Larvae_BTI_13	139360	116427	115914	116136	115263	104871	104871	103317	1554	98.52	1.48
Larvae_BTI_14	149082	128228	127677	127847	126806	109956	109956	109774	182	99.83	0.17
Larvae_BTI_15	133744	113178	112616	112858	111612	93820	93820	93666	154	99.84	0.16
Larvae_BTI_16	142614	120690	120349	120426	119444	104842	104842	101566	3276	96.88	3.12
Female_Control_1	161707	141775	141273	141181	140142	132158	132158	25377	106781	19.20	80.80
Female_Control_2	171358	152682	152161	151754	146947	140639	140639	10107	130532	7.19	92.81
Female_Control_3	126202	109215	108908	108907	108343	102184	102184	22725	79459	22.24	77.76
Female_Control_4	105661	88392	87978	88146	87295	81023	81023	26057	54966	32.16	67.84
Female_Control_5	144439	115532	114814	114844	112925	83724	83724	59856	23868	71.49	28.51
Female_Control_6	155046	139128	138787	138784	137588	131611	131611	19184	112427	14.58	85.42
Female_Control_7	134789	119091	118555	118714	117325	110890	110890	20141	90749	18.16	81.84
Female_Control_8	180705	149573	148959	148574	147241	129116	129116	86263	42853	66.81	33.19
Female_Control_9	138866	121772	121462	121379	120557	111880	111880	10990	100890	9.82	90.18
Female_Control_10	159643	138027	136997	137129	131098	116000	116000	43685	72315	37.66	62.34
Female_Control_11	178875	159988	159707	159673	159035	154448	154448	3642	150806	2.36	97.64
Female_Control_12	134726	119917	119471	119623	118665	115461	115461	6162	109299	5.34	94.66
Female_Control_13	115888	98249	97876	97795	97008	86889	86889	30487	56402	35.09	64.91

Female_Control_ 15	141093	121515	121089	121236	120418	117011	117011	8533	108478	7.29	92.71
Female_Control_ 16	123821	109529	109198	109207	108502	102510	102510	14501	88009	14.15	85.85
Female_BTI_1	152855	127357	126095	126177	113644	93649	93649	66662	26987	71.18	28.82
Female_BTI_2	166493	149215	148888	148671	147862	142355	142355	12226	130129	8.59	91.41
Female_BTI_3	138279	116174	115462	115472	112143	90003	90003	33800	56203	37.55	62.45
Female_BTI_4	144995	130107	129894	129957	129475	125843	125843	1630	124213	1.30	98.70
Female_BTI_5	133825	113745	113480	113518	113084	106684	106684	30537	76147	28.62	71.38
Female_BTI_6	152294	130902	130500	130462	129139	122354	122354	22664	99690	18.52	81.48
Female_BTI_7	144926	116140	115195	115134	107892	91555	91555	70476	21079	76.98	23.02
Female_BTI_8	118445	106696	106468	106394	105753	102329	102329	6286	96043	6.14	93.86
Female_BTI_9	158594	135498	134861	134719	132523	120015	120015	39457	80558	32.88	67.12
Female_BTI_10	163191	146676	146141	146309	145075	138477	138477	8006	130471	5.78	94.22
Female_BTI_11	161331	144498	144157	144210	143615	138763	138763	2192	136571	1.58	98.42
Female_BTI_12	169671	147911	147517	147632	146871	134141	134141	31190	102951	23.25	76.75
Female_BTI_13	167978	150744	150357	150424	149652	142593	142593	12486	130107	8.76	91.24
Female_BTI_14	158778	143335	142955	143058	142324	137270	137270	4243	133027	3.09	96.91
Female_BTI_15	129341	112680	112265	112472	111651	108332	108332	5660	102672	5.22	94.78
Female_BTI_16	170857	153491	153164	153176	152226	142918	142918	7782	135136	5.45	94.55
