# Supplementary material for: Carry-over effects of Bacillus thuringiensis on tolerant Aedes albopictus mosquitoes
Source: Parasit Vectors. 2024 Nov 7;17:456. doi: 10.1186/s13071-024-06556-3 (PMC11545555; doi:10.1186/s13071-024-06556-3)
Supplement: Supplementary file 10 — Additional file 10: Figure S4. Bar plot of the relative abundances of microbial community at the rank of phylum (A), family (B), and genus (C) in the gut of Bti-tolerant larvae and their derived adults and respective controls. Each bar in panels A and B represents the most abundant bacterial phylum and family, respectively, for each experimental group. Each bar in panel C shows the relative abundance assigned to a given bacterial genus, excluding Wolbachia, for each individual gut. [file 13071_2024_6556_MOESM10_ESM.pdf]

Additional File 7: Table S4. Average relative abundance of sequences assigned to a given bacterial taxon at genus level, in the LC, LB , AC and AB samples. The table includes a label indicating whether the Genus was identified (P) or not (A) in a group

Kingdom	Phylum	Class	Order	Family	Genus	Mean_Abundance_LC	SD_Abundance_LC	Mean_Abundance_LB	SD_Abundance_LB	Mean_Abundance_AC	SD_Abundance_AC	Mean_Abundance_AB	SD_Abundance_AB	AB	AC	LB	LC
Bacteria	Abditibacteriota	Abditibacteria	Abditibacteriales	Abditibacteriaceae	Abditibacterium	0.00	0.00	0.00	0.00	0.00	0.00	0.00	0.00	A	A	A	P
Bacteria	Acidobacteriota	Acidobacteriae	Paludibaculum	Unknown	Unknown	0.00	0.00	0.00	0.01	0.00	0.00	0.00	0.00	A	P	A	A
Bacteria	Acidobacteriota	Blastocatellia	11-24	Unknown	Unknown	0.00	0.00	0.00	0.00	0.00	0.00	0.00	0.00	P	A	A	A
Bacteria	Acidobacteriota	Blastocatellia	Blastocatellales	Blastocatellaceae	Stenotrophobacter	0.00	0.00	0.00	0.01	0.00	0.00	0.00	0.00	A	P	A	A
Bacteria	Acidobacteriota	Blastocatellia	Blastocatellales	Blastocatellaceae	Unknown	0.01	0.03	0.00	0.00	0.00	0.01	0.00	0.00	P	A	P	A
Bacteria	Acidobacteriota	Blastocatellia	DS-100	Unknown	Unknown	0.00	0.00	0.00	0.00	0.00	0.01	0.00	0.00	A	A	P	A
Bacteria	Acidobacteriota	Blastocatellia	Pyrinomonadales	Pyrinomonadaceae	RB41	0.00	0.00	0.00	0.00	0.00	0.00	0.00	0.01	A	A	A	P
Bacteria	Acidobacteriota	Vicinamibacteria	Vicinamibacterales	Vicinamibacteraceae	Unknown	0.00	0.00	0.00	0.00	0.00	0.02	0.00	0.00	A	A	P	A
Bacteria	Actinobacteriota	Acidimicrobiia	Microtrichales	Ilumatobacteraceae	Unknown	0.00	0.00	0.00	0.00	0.00	0.00	0.00	0.00	A	P	A	P
Bacteria	Actinobacteriota	Acidimicrobiia	Unknown	Unknown	Unknown	0.00	0.00	0.00	0.00	0.00	0.00	0.00	0.00	A	A	A	P
Bacteria	Actinobacteriota	Actinobacteria	Actinomycetales	Actinomycetaceae	Actinomyces	0.00	0.00	0.00	0.00	0.01	0.02	0.00	0.01	A	A	P	P
Bacteria	Actinobacteriota	Actinobacteria	Bifidobacteriales	Bifidobacteriaceae	Bifidobacterium	0.00	0.00	0.00	0.00	0.00	0.00	0.00	0.01	A	A	A	P
Bacteria	Actinobacteriota	Actinobacteria	Bifidobacteriales	Bifidobacteriaceae	Gardnerella	0.00	0.01	0.00	0.00	0.01	0.04	0.03	0.08	P	A	P	P
Bacteria	Actinobacteriota	Actinobacteria	Corynebacteriales	Corynebacteriaceae	Corynebacterium	0.00	0.01	0.01	0.02	0.04	0.05	0.11	0.17	P	P	P	P
Bacteria	Actinobacteriota	Actinobacteria	Corynebacteriales	Corynebacteriaceae	Lawsonella	0.00	0.01	0.02	0.04	0.03	0.04	0.09	0.19	P	P	P	P
Bacteria	Actinobacteriota	Actinobacteria	Corynebacteriales	Corynebacteriaceae	Turicella	0.00	0.00	0.00	0.00	0.00	0.00	0.00	0.02	A	A	A	P
Bacteria	Actinobacteriota	Actinobacteria	Corynebacteriales	Dietziaceae	Dietzia	0.00	0.00	0.00	0.00	0.00	0.00	0.00	0.01	A	A	A	P
Bacteria	Actinobacteriota	Actinobacteria	Corynebacteriales	Mycobacteriaceae	Mycobacterium	0.00	0.01	0.01	0.01	0.00	0.01	0.00	0.01	P	P	P	P
Bacteria	Actinobacteriota	Actinobacteria	Corynebacteriales	Nocardiaceae	Gordonia	0.00	0.00	0.00	0.00	0.00	0.00	0.00	0.01	A	A	P	P
Bacteria	Actinobacteriota	Actinobacteria	Corynebacteriales	Nocardiaceae	Nocardia	0.00	0.00	0.00	0.00	0.00	0.00	0.00	0.01	A	A	P	P
Bacteria	Actinobacteriota	Actinobacteria	Corynebacteriales	Nocardiaceae	Rhodococcus	0.01	0.02	0.00	0.01	0.05	0.08	0.09	0.14	P	P	P	P
Bacteria	Actinobacteriota	Actinobacteria	Frankiales	Geodermatophilaceae	Klenkia	0.00	0.00	0.00	0.00	0.00	0.01	0.00	0.00	A	A	P	A
Bacteria	Actinobacteriota	Actinobacteria	Frankiales	Sporichthyaceae	hgcI clade	0.00	0.00	0.00	0.00	0.00	0.00	0.01	0.03	A	A	A	P
Bacteria	Actinobacteriota	Actinobacteria	Kineosporiales	Kineosporiaceae	Quadrisphaera	0.00	0.00	0.00	0.00	0.01	0.05	0.00	0.00	A	A	P	A

Bacteria	Actinobacteriota	Actinobacteria	Micrococcales	Beutenbergiaceae	Miniimonas	1.19	1.19	1.82	3.38	0.00	0.00	0.00	0.00	P	P	A	A
Bacteria	Actinobacteriota	Actinobacteria	Micrococcales	Beutenbergiaceae	Unknown	0.00	0.00	0.00	0.00	0.00	0.00	0.00	0.00	P	A	A	A
Bacteria	Actinobacteriota	Actinobacteria	Micrococcales	Brevibacteriaceae	Brevibacterium	0.00	0.00	0.00	0.01	0.01	0.03	0.01	0.01	A	P	P	P
Bacteria	Actinobacteriota	Actinobacteria	Micrococcales	Dermabacteraceae	Brachybacterium	0.00	0.00	0.00	0.00	0.00	0.01	0.00	0.01	A	A	P	P
Bacteria	Actinobacteriota	Actinobacteria	Micrococcales	Dermacoccaceae	Dermacoccus	0.00	0.00	0.00	0.00	0.00	0.01	0.01	0.02	A	A	P	P
Bacteria	Actinobacteriota	Actinobacteria	Micrococcales	Intrasporangiaceae	Knoellia	0.00	0.00	0.00	0.00	0.00	0.01	0.00	0.02	A	A	P	P
Bacteria	Actinobacteriota	Actinobacteria	Micrococcales	Intrasporangiaceae	Ornithinibacter	0.00	0.00	0.00	0.00	0.00	0.00	0.00	0.00	A	P	A	A
Bacteria	Actinobacteriota	Actinobacteria	Micrococcales	Intrasporangiaceae	Terrabacter	0.00	0.00	0.00	0.00	0.00	0.00	0.00	0.00	A	A	A	P
Bacteria	Actinobacteriota	Actinobacteria	Micrococcales	Microbacteriaceae	Curtobacterium	0.00	0.00	0.00	0.00	0.00	0.00	0.00	0.00	A	A	A	P
Bacteria	Actinobacteriota	Actinobacteria	Micrococcales	Microbacteriaceae	Galbitalea	0.04	0.06	0.03	0.05	0.00	0.00	0.00	0.00	P	P	A	A
Bacteria	Actinobacteriota	Actinobacteria	Micrococcales	Microbacteriaceae	Leifsonia	0.00	0.00	0.03	0.05	0.00	0.00	0.00	0.00	A	P	A	A
Bacteria	Actinobacteriota	Actinobacteria	Micrococcales	Microbacteriaceae	Microbacterium	82.57	6.75	48.27	27.47	0.05	0.09	0.07	0.17	P	P	P	P
Bacteria	Actinobacteriota	Actinobacteria	Micrococcales	Microbacteriaceae	Microterricola	0.00	0.00	0.00	0.00	0.00	0.00	0.00	0.01	A	A	A	P
Bacteria	Actinobacteriota	Actinobacteria	Micrococcales	Micrococcaceae	Kocuria	0.00	0.00	0.00	0.00	0.01	0.02	0.02	0.04	P	A	P	P
Bacteria	Actinobacteriota	Actinobacteria	Micrococcales	Micrococcaceae	Micrococcus	0.00	0.00	0.00	0.00	0.00	0.01	0.01	0.03	A	A	P	P
Bacteria	Actinobacteriota	Actinobacteria	Micrococcales	Micrococcaceae	Renibacterium	0.00	0.00	0.00	0.00	0.00	0.00	0.00	0.01	A	A	A	P
Bacteria	Actinobacteriota	Actinobacteria	Micrococcales	Micrococcaceae	Rothia	0.00	0.00	0.00	0.00	0.02	0.09	0.00	0.00	P	A	P	A
Bacteria	Actinobacteriota	Actinobacteria	Micromonosporales	Micromonosporaceae	Verrucosispora	0.00	0.00	0.00	0.00	0.00	0.00	0.00	0.01	A	A	A	P
Bacteria	Actinobacteriota	Actinobacteria	Propionibacteriales	Nocardioidaceae	Nocardioides	0.00	0.00	0.01	0.03	0.00	0.01	0.42	1.42	A	P	P	P
Bacteria	Actinobacteriota	Actinobacteria	Propionibacteriales	Propionibacteriaceae	Cutibacterium	0.01	0.01	0.03	0.03	0.08	0.09	0.11	0.14	P	P	P	P
Bacteria	Actinobacteriota	Actinobacteria	Pseudonocardiales	Pseudonocardiaceae	Pseudonocardia	0.00	0.00	0.00	0.00	0.00	0.00	0.00	0.00	A	A	P	A
Bacteria	Actinobacteriota	Rubrobacteria	Rubrobacterales	Rubrobacteriaceae	Rubrobacter	0.00	0.00	0.00	0.00	0.00	0.00	0.00	0.00	P	A	A	A
Bacteria	Actinobacteriota	Thermoleophilia	Gaiellales	Gaiellaceae	Gaiella	0.00	0.00	0.00	0.00	0.01	0.03	0.00	0.00	A	A	P	A
Bacteria	Actinobacteriota	Thermoleophilia	Solirubrobacterales	67-14	Unknown	0.00	0.00	0.00	0.00	0.00	0.00	0.00	0.00	A	P	P	P
Bacteria	Actinobacteriota	Thermoleophilia	Solirubrobacterales	Solirubrobacteraceae	Conexibacter	0.00	0.00	0.00	0.00	0.00	0.00	0.00	0.00	A	A	P	A
Bacteria	Actinobacteriota	Thermoleophilia	Solirubrobacterales	Solirubrobacteraceae	Solirubrobacter	0.00	0.00	0.00	0.00	0.00	0.01	0.00	0.01	A	A	P	P
Bacteria	Actinobacteriota	Thermoleophilia	Solirubrobacterales	Unknown	Unknown	0.00	0.00	0.00	0.00	0.00	0.00	0.01	0.03	A	A	A	P

Bacteria	Armatimonadota	Unknown	Unknown	Unknown	Unknown	0.00	0.00	0.00	0.00	0.00	0.00	0.00	0.01	A	A	A	P
Bacteria	Bacteroidota	Bacteroidia	Bacteroidales	Bacteroidaceae	Bacteroides	0.00	0.00	0.00	0.00	0.02	0.05	0.01	0.06	A	A	P	P
Bacteria	Bacteroidota	Bacteroidia	Bacteroidales	Barnesiellaceae	Coprobacter	0.00	0.00	0.00	0.00	0.01	0.03	0.00	0.00	A	A	P	A
Bacteria	Bacteroidota	Bacteroidia	Bacteroidales	Dysgonomonadaceae	Unknown	0.00	0.00	0.00	0.00	0.00	0.01	0.00	0.00	A	A	P	A
Bacteria	Bacteroidota	Bacteroidia	Bacteroidales	Marinifilaceae	Odoribacter	0.00	0.00	0.00	0.00	0.00	0.00	0.00	0.00	P	A	A	A
Bacteria	Bacteroidota	Bacteroidia	Bacteroidales	Muribaculaceae	Unknown	0.00	0.00	0.00	0.00	0.01	0.03	0.00	0.00	A	A	P	A
Bacteria	Bacteroidota	Bacteroidia	Bacteroidales	Porphyromonadaceae	Porphyromonas	0.00	0.00	0.00	0.00	0.01	0.02	0.00	0.01	A	A	P	P
Bacteria	Bacteroidota	Bacteroidia	Bacteroidales	Prevotellaceae	Alloprevotella	0.00	0.00	0.00	0.00	0.00	0.02	0.00	0.00	A	A	P	A
Bacteria	Bacteroidota	Bacteroidia	Bacteroidales	Prevotellaceae	Prevotella	0.00	0.00	0.00	0.01	0.00	0.00	0.01	0.03	A	P	P	P
Bacteria	Bacteroidota	Bacteroidia	Bacteroidales	Prevotellaceae	Prevotella_7	0.00	0.00	0.00	0.00	0.01	0.02	0.00	0.00	A	A	P	A
Bacteria	Bacteroidota	Bacteroidia	Bacteroidales	Prevotellaceae	Prevotella_9	0.00	0.00	0.00	0.01	0.01	0.03	0.00	0.01	A	P	P	P
Bacteria	Bacteroidota	Bacteroidia	Chitinophagales	Chitinophagaceae	Asinibacterium	0.00	0.00	0.00	0.01	0.00	0.00	0.01	0.03	A	P	P	P
Bacteria	Bacteroidota	Bacteroidia	Chitinophagales	Chitinophagaceae	Edaphobaculum	0.00	0.00	0.00	0.00	0.00	0.00	0.00	0.00	P	A	A	A
Bacteria	Bacteroidota	Bacteroidia	Chitinophagales	Chitinophagaceae	Flavisolibacter	0.00	0.00	0.00	0.00	0.00	0.02	0.00	0.00	A	A	P	A
Bacteria	Bacteroidota	Bacteroidia	Chitinophagales	Chitinophagaceae	Niabella	0.00	0.00	0.00	0.00	0.00	0.00	0.02	0.04	A	A	A	P
Bacteria	Bacteroidota	Bacteroidia	Chitinophagales	Chitinophagaceae	Puia	0.00	0.00	0.00	0.00	0.01	0.03	0.00	0.00	A	A	P	A
Bacteria	Bacteroidota	Bacteroidia	Cytophagales	Cytophagaceae	Siphonobacter	0.00	0.00	0.06	0.10	0.05	0.13	0.00	0.01	P	P	P	P
Bacteria	Bacteroidota	Bacteroidia	Cytophagales	Hymenobacteraceae	Hymenobacter	0.00	0.00	0.00	0.00	0.01	0.01	0.01	0.02	A	A	P	P
Bacteria	Bacteroidota	Bacteroidia	Cytophagales	Hymenobacteraceae	Nibribacter	0.00	0.00	0.00	0.00	0.00	0.01	0.00	0.00	A	A	P	A
Bacteria	Bacteroidota	Bacteroidia	Cytophagales	Microscillaceae	Ohtaekwangia	0.00	0.00	0.00	0.00	0.00	0.02	0.00	0.00	A	A	P	A
Bacteria	Bacteroidota	Bacteroidia	Cytophagales	Microscillaceae	Unknown	0.00	0.00	0.00	0.00	0.00	0.00	0.00	0.01	A	A	A	P
Bacteria	Bacteroidota	Bacteroidia	Cytophagales	Spirosomaceae	Dyadobacter	0.00	0.00	0.00	0.01	0.00	0.00	0.00	0.00	A	P	P	A
Bacteria	Bacteroidota	Bacteroidia	Cytophagales	Spirosomaceae	Fibrisoma	0.00	0.00	0.00	0.00	0.00	0.00	0.02	0.04	A	A	A	P
Bacteria	Bacteroidota	Bacteroidia	Cytophagales	Spirosomaceae	Spirosoma	0.00	0.00	0.00	0.00	0.00	0.01	0.00	0.00	A	A	P	A
Bacteria	Bacteroidota	Bacteroidia	Flavobacteriales	Flavobacteriaceae	Aquibacter	0.00	0.00	0.00	0.00	0.00	0.01	0.00	0.00	A	A	P	A
Bacteria	Bacteroidota	Bacteroidia	Flavobacteriales	Flavobacteriaceae	Capnocytophaga	0.00	0.00	0.00	0.00	0.00	0.01	0.00	0.00	A	A	P	A
Bacteria	Bacteroidota	Bacteroidia	Flavobacteriales	Flavobacteriaceae	Flavobacterium	0.00	0.00	0.00	0.00	0.01	0.03	0.00	0.00	A	A	P	A

Bacteria	Bacteroidota	Bacteroidia	Flavobacteriales	Weeksellaceae	Chryseobacterium	0.02	0.03	0.39	0.92	0.52	1.86	0.42	1.16	P	P	P	P
Bacteria	Bacteroidota	Bacteroidia	Flavobacteriales	Weeksellaceae	Cloacibacterium	0.00	0.00	0.00	0.01	0.02	0.04	0.01	0.03	A	P	P	P
Bacteria	Bacteroidota	Bacteroidia	Flavobacteriales	Weeksellaceae	Empedobacter	0.00	0.00	0.00	0.00	0.01	0.02	0.01	0.03	A	A	P	P
Bacteria	Bacteroidota	Bacteroidia	Flavobacteriales	Weeksellaceae	Weeksella	0.00	0.01	0.00	0.00	0.00	0.00	0.00	0.01	P	A	A	P
Bacteria	Bacteroidota	Bacteroidia	Sphingobacteriales	Sphingobacteriaceae	Nubsella	0.00	0.00	0.00	0.00	0.00	0.02	0.00	0.00	A	A	P	A
Bacteria	Bacteroidota	Bacteroidia	Sphingobacteriales	Sphingobacteriaceae	Pedobacter	0.00	0.01	0.00	0.01	0.40	1.48	0.95	3.39	P	P	P	P
Bacteria	Bacteroidota	Bacteroidia	Sphingobacteriales	Sphingobacteriaceae	Sphingobacterium	0.00	0.00	0.00	0.00	0.00	0.01	0.09	0.33	A	A	P	P
Bacteria	Bacteroidota	Bacteroidia	Sphingobacteriales	Sphingobacteriaceae	Unknown	0.00	0.00	0.00	0.00	0.01	0.04	0.00	0.00	A	A	P	A
Bacteria	Bacteroidota	Bacteroidia	Sphingobacteriales	env.OPS 17	Unknown	0.00	0.00	0.00	0.00	0.00	0.00	0.00	0.01	P	A	A	P
Bacteria	Bdellovibrionota	Bdellovibrionia	Bacteriovoracales	Bacteriovoracaceae	Peredibacter	0.00	0.00	0.62	1.40	0.00	0.01	0.00	0.00	A	P	P	A
Bacteria	Campylobacterota	Campylobacteria	Campylobacterales	Campylobacteraceae	Campylobacter	0.00	0.00	0.00	0.00	0.00	0.00	0.00	0.02	A	A	A	P
Bacteria	Cyanobacteria	Cyanobacteriia	Unknown	Unknown	Unknown	0.00	0.00	0.00	0.00	0.00	0.00	0.00	0.00	A	A	P	A
Bacteria	Deinococcota	Deinococci	Deinococcales	Deinococcaceae	Deinococcus	0.00	0.00	0.00	0.00	0.01	0.02	0.00	0.00	A	A	P	A
Bacteria	Deinococcota	Deinococci	Thermales	Thermaceae	Thermus	0.00	0.00	0.00	0.00	0.00	0.00	0.00	0.00	A	P	A	A
Bacteria	Desulfobacterota	Desulfomonilia	Desulfomonilales	Desulfomonilaceae	Desulfomonile	0.00	0.00	0.00	0.00	0.00	0.00	0.00	0.00	A	P	A	A
Bacteria	Firmicutes	Bacilli	Bacillales	Bacillaceae	Bacillus	0.00	0.00	4.65	5.69	0.02	0.04	0.02	0.03	P	P	P	P
Bacteria	Firmicutes	Bacilli	Bacillales	Bacillaceae	Geobacillus	0.42	0.68	4.04	7.21	9.98	15.12	10.92	16.12	P	P	P	P
Bacteria	Firmicutes	Bacilli	Bacillales	Planococcaceae	Lysinibacillus	0.00	0.00	0.00	0.00	0.00	0.00	0.01	0.02	A	A	A	P
Bacteria	Firmicutes	Bacilli	Bacillales	Planococcaceae	Planococcus	0.00	0.00	0.00	0.00	0.00	0.00	0.00	0.00	A	P	A	A
Bacteria	Firmicutes	Bacilli	Brevibacillales	Brevibacillaceae	Brevibacillus	0.07	0.16	0.16	0.28	0.69	1.02	0.20	0.40	P	P	P	P
Bacteria	Firmicutes	Bacilli	Lactobacillales	Aerococcaceae	Aerococcus	0.00	0.00	0.00	0.00	0.00	0.00	0.00	0.02	A	A	P	P
Bacteria	Firmicutes	Bacilli	Lactobacillales	Aerococcaceae	Ignavigranum	0.00	0.00	0.00	0.00	0.00	0.00	0.00	0.01	A	A	A	P
Bacteria	Firmicutes	Bacilli	Lactobacillales	Carnobacteriaceae	Granulicatella	0.00	0.00	0.00	0.00	0.02	0.04	0.00	0.00	A	P	P	A
Bacteria	Firmicutes	Bacilli	Lactobacillales	Enterococcaceae	Enterococcus	0.00	0.00	0.00	0.00	0.00	0.00	0.00	0.00	A	A	A	P
Bacteria	Firmicutes	Bacilli	Lactobacillales	Lactobacillaceae	Bombilactobacillus	0.00	0.00	0.00	0.00	0.00	0.01	0.00	0.00	A	A	P	A
Bacteria	Firmicutes	Bacilli	Lactobacillales	Lactobacillaceae	HT002	0.00	0.00	0.00	0.00	0.00	0.01	0.00	0.00	A	A	P	A
Bacteria	Firmicutes	Bacilli	Lactobacillales	Lactobacillaceae	Lactobacillus	0.02	0.02	0.00	0.02	0.04	0.05	0.04	0.07	P	P	P	P

Bacteria	Firmicutes	Bacilli	Lactobacillales	Lactobacillaceae	Latilactobacillus	0.00	0.00	0.00	0.00	0.00	0.00	0.00	0.02	A	A	A	P
Bacteria	Firmicutes	Bacilli	Lactobacillales	Lactobacillaceae	Leuconostoc	0.00	0.00	0.00	0.00	0.00	0.00	0.00	0.02	A	A	A	P
Bacteria	Firmicutes	Bacilli	Lactobacillales	Streptococcaceae	Lactococcus	0.00	0.00	0.00	0.00	0.00	0.00	0.00	0.00	P	A	A	A
Bacteria	Firmicutes	Bacilli	Lactobacillales	Streptococcaceae	Streptococcus	0.00	0.00	0.01	0.03	0.06	0.11	0.09	0.16	P	P	P	P
Bacteria	Firmicutes	Bacilli	Paenibacillales	Paenibacillaceae	Paenibacillus	0.00	0.00	0.00	0.01	0.00	0.00	0.00	0.00	A	P	A	A
Bacteria	Firmicutes	Bacilli	Staphylococcales	Gemellaceae	Gemella	0.00	0.00	0.00	0.01	0.01	0.03	0.00	0.00	A	P	P	P
Bacteria	Firmicutes	Bacilli	Staphylococcales	Staphylococcaceae	Jeotgalicoccus	0.00	0.00	0.00	0.00	0.00	0.00	0.00	0.01	A	A	A	P
Bacteria	Firmicutes	Bacilli	Staphylococcales	Staphylococcaceae	Staphylococcus	0.10	0.09	0.18	0.16	0.75	0.94	0.86	1.11	P	P	P	P
Bacteria	Firmicutes	Clostridia	Christensenellales	Christensenellaceae	Unknown	0.00	0.00	0.00	0.00	0.00	0.01	0.00	0.00	A	A	P	A
Bacteria	Firmicutes	Clostridia	Clostridia UCG-014	Unknown	Unknown	0.00	0.00	0.00	0.00	0.00	0.00	0.00	0.02	A	A	A	P
Bacteria	Firmicutes	Clostridia	Clostridiales	Clostridiaceae	Clostridium sensu stricto 1	0.00	0.00	0.03	0.04	0.01	0.02	0.01	0.02	A	P	P	P
Bacteria	Firmicutes	Clostridia	Lachnospirales	Lachnospiraceae	A2	0.00	0.00	0.00	0.00	0.00	0.01	0.00	0.00	A	A	P	A
Bacteria	Firmicutes	Clostridia	Lachnospirales	Lachnospiraceae	Blautia	0.00	0.00	0.00	0.02	0.00	0.00	0.00	0.02	A	P	A	P
Bacteria	Firmicutes	Clostridia	Lachnospirales	Lachnospiraceae	Coprococcus	0.00	0.00	0.00	0.00	0.00	0.00	0.01	0.02	A	A	A	P
Bacteria	Firmicutes	Clostridia	Lachnospirales	Lachnospiraceae	Fusicatenibacter	0.00	0.00	0.00	0.00	0.00	0.00	0.01	0.04	A	A	A	P
Bacteria	Firmicutes	Clostridia	Lachnospirales	Lachnospiraceae	GCA-900066575	0.00	0.00	0.00	0.00	0.00	0.01	0.00	0.00	A	A	P	A
Bacteria	Firmicutes	Clostridia	Lachnospirales	Lachnospiraceae	Herbinix	0.00	0.00	0.00	0.00	0.00	0.00	0.00	0.00	A	A	P	A
Bacteria	Firmicutes	Clostridia	Lachnospirales	Lachnospiraceae	Johnsonella	0.00	0.00	0.00	0.00	0.01	0.03	0.00	0.00	A	A	P	A
Bacteria	Firmicutes	Clostridia	Lachnospirales	Lachnospiraceae	Lachnoanaerobaculum	0.00	0.00	0.00	0.00	0.00	0.00	0.00	0.00	A	P	P	A
Bacteria	Firmicutes	Clostridia	Lachnospirales	Lachnospiraceae	Lachnospiraceae NC2004 group	0.00	0.00	0.00	0.00	0.00	0.02	0.00	0.00	A	A	P	A
Bacteria	Firmicutes	Clostridia	Lachnospirales	Lachnospiraceae	Lachnospiraceae NK4A136 group	0.00	0.00	0.00	0.00	0.00	0.00	0.01	0.04	A	A	A	P
Bacteria	Firmicutes	Clostridia	Lachnospirales	Lachnospiraceae	Roseburia	0.00	0.00	0.00	0.00	0.00	0.00	0.00	0.00	P	A	A	A
Bacteria	Firmicutes	Clostridia	Lachnospirales	Lachnospiraceae	Unknown	0.00	0.00	0.00	0.01	0.00	0.01	0.01	0.05	A	P	P	P
Bacteria	Firmicutes	Clostridia	Oscillospirales	Hungateiclostridiaceae	Ruminiclostridium	0.00	0.00	0.00	0.00	0.00	0.00	0.00	0.01	A	A	A	P
Bacteria	Firmicutes	Clostridia	Oscillospirales	Oscillospiraceae	UCG-005	0.00	0.00	0.00	0.00	0.00	0.00	0.00	0.00	A	P	A	A

Bacteria	Firmicutes	Clostridia	Oscillospirales	Ruminococcaceae	Faecalibacterium	0.00	0.00	0.00	0.00	0.00	0.00	0.02	0.06	A	A	A	P
Bacteria	Firmicutes	Clostridia	Oscillospirales	Ruminococcaceae	Subdoligranulum	0.00	0.00	0.00	0.00	0.00	0.00	0.03	0.14	A	A	A	P
Bacteria	Firmicutes	Clostridia	Oscillospirales	Ruminococcaceae	Unknown	0.00	0.00	0.00	0.00	0.00	0.01	0.00	0.00	A	A	P	A
Bacteria	Firmicutes	Clostridia	Peptostreptococcales-Tissierellales	Anaerovoracaceae	[Eubacterium] nodatum group	0.00	0.00	0.00	0.00	0.00	0.00	0.00	0.00	A	A	P	A
Bacteria	Firmicutes	Clostridia	Peptostreptococcales-Tissierellales	Family XI	Anaerococcus	0.00	0.00	0.00	0.00	0.00	0.00	0.01	0.04	A	A	A	P
Bacteria	Firmicutes	Clostridia	Peptostreptococcales-Tissierellales	Family XI	Finegoldia	0.00	0.00	0.00	0.00	0.01	0.02	0.02	0.05	P	P	P	P
Bacteria	Firmicutes	Clostridia	Peptostreptococcales-Tissierellales	Family XI	Peptoniphilus	0.00	0.00	0.00	0.00	0.00	0.01	0.01	0.03	P	A	P	P
Bacteria	Firmicutes	Clostridia	Peptostreptococcales-Tissierellales	Peptostreptococcaceae	Paeniclostridium	0.00	0.00	0.00	0.00	0.00	0.00	0.00	0.02	A	A	A	P
Bacteria	Firmicutes	Clostridia	Peptostreptococcales-Tissierellales	Peptostreptococcaceae	Romboutsia	0.00	0.00	0.00	0.00	0.00	0.00	0.00	0.01	A	A	A	P
Bacteria	Firmicutes	Clostridia	Peptostreptococcales-Tissierellales	Peptostreptococcaceae	Sporacetigenium	0.00	0.00	0.00	0.00	0.00	0.01	0.00	0.00	A	A	P	A
Bacteria	Firmicutes	Negativicutes	Acidaminococcales	Acidaminococcaceae	Succiniclasticum	0.00	0.00	0.00	0.00	0.00	0.02	0.00	0.00	A	A	P	A
Bacteria	Firmicutes	Negativicutes	Veillonellales-Selenomonadales	Selenomonadaceae	Centipeda	0.00	0.00	0.00	0.00	0.00	0.00	0.00	0.00	A	P	A	A
Bacteria	Firmicutes	Negativicutes	Veillonellales-Selenomonadales	Selenomonadaceae	Mitsuokella	0.00	0.00	0.00	0.00	0.00	0.01	0.00	0.00	A	A	P	A
Bacteria	Firmicutes	Negativicutes	Veillonellales-Selenomonadales	Veillonellaceae	Veillonella	0.00	0.00	0.00	0.00	0.01	0.03	0.00	0.01	A	A	P	P
Bacteria	Firmicutes	Thermoanaerobacteria	Thermoanaerobacterales	Family III	Thermoanaerobacterium	0.00	0.00	0.00	0.00	0.00	0.00	0.00	0.02	A	A	A	P
Bacteria	Fusobacteriota	Fusobacteriia	Fusobacteriales	Fusobacteriaceae	Fusobacterium	0.00	0.00	0.00	0.00	0.00	0.02	0.00	0.00	A	A	P	A
Bacteria	Fusobacteriota	Fusobacteriia	Fusobacteriales	Leptotrichiaceae	Leptotrichia	0.00	0.00	0.00	0.00	0.00	0.00	0.00	0.00	A	P	A	A
Bacteria	Gemmatimonadota	Gemmatimonadetes	Gemmatimonadales	Gemmatimonadaceae	Unknown	0.00	0.00	0.00	0.00	0.00	0.01	0.00	0.00	A	A	P	A
Bacteria	MBNT15	Unknown	Unknown	Unknown	Unknown	0.00	0.00	0.00	0.00	0.00	0.00	0.02	0.08	A	A	A	P
Bacteria	Myxococcota	Myxococcia	Myxococcales	Myxococcaceae	P3OB-42	0.00	0.00	0.00	0.00	0.00	0.01	0.00	0.00	A	A	P	A
Bacteria	Myxococcota	Polyangia	Haliangiales	Haliangiaceae	Haliangium	0.00	0.00	0.00	0.00	0.00	0.02	0.00	0.00	A	A	P	A

Bacteria	Myxococcota	Polyangia	Polyangiales	Phaselicystidaceae	Phaselicystis	0.00	0.00	0.00	0.00	0.00	0.00	0.00	0.02	A	A	A	P
Bacteria	Myxococcota	Polyangia	Polyangiales	Polyangiaceae	Pajaroellobacter	0.00	0.00	0.00	0.00	0.00	0.01	0.00	0.00	A	A	P	A
Bacteria	Patescibacteria	Parcubacteria	Unknown	Unknown	Unknown	0.00	0.00	0.00	0.00	0.00	0.02	0.00	0.00	A	A	P	A
Bacteria	Patescibacteria	Saccharimonadia	Saccharimonadales	Saccharimonadaceae	Candidatus Saccharimonas	0.00	0.00	0.00	0.00	0.00	0.01	0.00	0.00	A	A	P	A
Bacteria	Patescibacteria	Saccharimonadia	Saccharimonadales	Saccharimonadaceae	TM7a	0.00	0.00	0.00	0.00	0.00	0.00	0.00	0.00	P	A	A	A
Bacteria	Patescibacteria	Saccharimonadia	Saccharimonadales	Unknown	Unknown	0.00	0.00	0.00	0.00	0.01	0.04	0.00	0.00	A	A	P	A
Bacteria	Planctomycetota	Phycisphaerae	Tepidisphaerales	Tepidisphaeraceae	Unknown	0.00	0.00	0.00	0.00	0.00	0.00	0.00	0.00	A	P	A	A
Bacteria	Planctomycetota	Phycisphaerae	Tepidisphaerales	WD2101 soil group	Unknown	0.00	0.00	0.00	0.00	0.00	0.00	0.00	0.01	A	A	A	P
Bacteria	Planctomycetota	Planctomycetes	Gemmatales	Gemmataceae	Fimbriiglobus	0.00	0.00	0.00	0.00	0.00	0.00	0.00	0.00	A	A	P	A
Bacteria	Planctomycetota	Planctomycetes	Gemmatales	Gemmataceae	Unknown	0.00	0.00	0.00	0.00	0.00	0.01	0.00	0.00	A	A	P	A
Bacteria	Planctomycetota	Planctomycetes	Isosphaerales	Isosphaeraceae	Tundrisphaera	0.00	0.00	0.00	0.00	0.00	0.02	0.00	0.00	A	A	P	A
Bacteria	Planctomycetota	Planctomycetes	Isosphaerales	Isosphaeraceae	Unknown	0.00	0.00	0.00	0.00	0.00	0.00	0.00	0.00	A	A	A	P
Bacteria	Planctomycetota	Planctomycetes	Pirellulales	Pirellulaceae	Unknown	0.00	0.00	0.00	0.00	0.00	0.00	0.00	0.00	A	P	A	A
Bacteria	Proteobacteria	Alphaproteobacteria	Acetobacterales	Acetobacteraceae	Acidiphilium	0.00	0.00	0.00	0.00	0.00	0.02	0.00	0.00	A	A	P	A
Bacteria	Proteobacteria	Alphaproteobacteria	Acetobacterales	Acetobacteraceae	Bombella	0.00	0.00	0.00	0.00	0.00	0.00	0.00	0.01	A	A	A	P
Bacteria	Proteobacteria	Alphaproteobacteria	Acetobacterales	Acetobacteraceae	Craurococcus-Caldovatus	0.00	0.00	0.00	0.00	0.01	0.04	0.00	0.00	A	A	P	A
Bacteria	Proteobacteria	Alphaproteobacteria	Acetobacterales	Acetobacteraceae	Roseomonas	0.00	0.00	0.00	0.00	0.00	0.02	0.00	0.00	P	A	P	A
Bacteria	Proteobacteria	Alphaproteobacteria	Acetobacterales	Acetobacteraceae	Rubritepida	4.72	3.83	0.97	0.85	0.00	0.01	0.00	0.00	P	P	P	A
Bacteria	Proteobacteria	Alphaproteobacteria	Acetobacterales	Acetobacteraceae	Tanticharoenia	0.00	0.00	0.00	0.00	1.57	3.59	0.35	0.63	A	A	P	P
Bacteria	Proteobacteria	Alphaproteobacteria	Azospirillales	Azospirillaceae	Azospirillum	0.00	0.00	0.00	0.00	0.00	0.01	0.00	0.00	A	A	P	A
Bacteria	Proteobacteria	Alphaproteobacteria	Caulobacterales	Caulobacteraceae	Asticcacaulis	0.00	0.00	0.00	0.00	0.00	0.01	0.00	0.00	A	A	P	A
Bacteria	Proteobacteria	Alphaproteobacteria	Caulobacterales	Caulobacteraceae	Brevundimonas	0.01	0.02	0.00	0.00	0.04	0.06	0.01	0.03	P	P	P	P
Bacteria	Proteobacteria	Alphaproteobacteria	Caulobacterales	Caulobacteraceae	Caulobacter	0.00	0.00	0.00	0.00	0.00	0.00	0.00	0.00	A	A	P	A
Bacteria	Proteobacteria	Alphaproteobacteria	Caulobacterales	Caulobacteraceae	Unknown	0.00	0.00	0.00	0.00	0.01	0.01	0.00	0.01	A	A	P	P
Bacteria	Proteobacteria	Alphaproteobacteria	Defluviicoccales	Defluviicoccaceae	Defluviicoccus	0.00	0.00	0.00	0.00	0.00	0.00	0.00	0.00	A	A	A	P
Bacteria	Proteobacteria	Alphaproteobacteria	Holosporales	Holosporaceae	Candidatus Paraholospora	0.00	0.00	0.00	0.00	0.00	0.00	0.00	0.02	P	A	A	P

Bacteria	Proteobacteria	Alphaproteobacteria	Micavibrionales	Unknown	Unknown	0.00	0.00	0.00	0.00	0.00	0.02	0.00	0.01	A	A	P	P
Bacteria	Proteobacteria	Alphaproteobacteria	NRL2	Unknown	Unknown	0.00	0.00	0.00	0.01	0.00	0.00	0.00	0.00	A	P	A	A
Bacteria	Proteobacteria	Alphaproteobacteria	Paracaedibacterales	Paracaedibacteraceae	Candidatus Paracaedibacter	0.00	0.00	0.00	0.00	0.00	0.00	0.00	0.00	A	A	A	P
Bacteria	Proteobacteria	Alphaproteobacteria	Reyranellales	Reyranellaceae	Reyranella	0.00	0.00	0.00	0.01	0.00	0.00	0.00	0.00	A	P	A	A
Bacteria	Proteobacteria	Alphaproteobacteria	Rhizobiales	Beijerinckiaceae	1174-901-12	0.00	0.00	0.00	0.00	0.05	0.20	0.00	0.00	A	A	P	A
Bacteria	Proteobacteria	Alphaproteobacteria	Rhizobiales	Beijerinckiaceae	28-YEA-48	0.00	0.00	0.00	0.01	0.00	0.00	0.00	0.00	A	P	A	A
Bacteria	Proteobacteria	Alphaproteobacteria	Rhizobiales	Beijerinckiaceae	Bosea	0.01	0.02	0.46	0.97	0.04	0.10	0.00	0.01	P	P	P	P
Bacteria	Proteobacteria	Alphaproteobacteria	Rhizobiales	Beijerinckiaceae	Methylobacterium-Methylorubrum	0.14	0.21	0.58	0.71	0.15	0.22	0.17	0.27	P	P	P	P
Bacteria	Proteobacteria	Alphaproteobacteria	Rhizobiales	Devosiaceae	Devosia	0.00	0.00	0.01	0.02	0.00	0.01	0.00	0.00	A	P	P	A
Bacteria	Proteobacteria	Alphaproteobacteria	Rhizobiales	Devosiaceae	Unknown	0.00	0.00	0.00	0.00	0.00	0.00	0.00	0.00	A	A	P	A
Bacteria	Proteobacteria	Alphaproteobacteria	Rhizobiales	Hyphomicrobiaceae	Pedomicrobium	0.00	0.00	0.00	0.00	0.00	0.01	0.00	0.00	A	A	P	A
Bacteria	Proteobacteria	Alphaproteobacteria	Rhizobiales	Kaistiaceae	Kaistia	8.33	4.76	18.65	17.66	0.00	0.01	0.00	0.00	P	P	P	A
Bacteria	Proteobacteria	Alphaproteobacteria	Rhizobiales	Rhizobiaceae	Allorhizobium-Neorhizobium-Pararhizobium-Rhizobium	0.05	0.07	0.53	1.52	0.70	1.62	0.02	0.04	P	P	P	P
Bacteria	Proteobacteria	Alphaproteobacteria	Rhizobiales	Rhizobiaceae	Aminobacter	0.00	0.00	0.28	0.90	0.00	0.00	0.00	0.00	A	P	A	A
Bacteria	Proteobacteria	Alphaproteobacteria	Rhizobiales	Rhizobiaceae	Aureimonas	0.00	0.00	0.00	0.00	0.00	0.01	0.01	0.02	P	A	P	P
Bacteria	Proteobacteria	Alphaproteobacteria	Rhizobiales	Rhizobiaceae	Mesorhizobium	0.00	0.00	0.09	0.27	0.05	0.11	0.00	0.01	A	P	P	P
Bacteria	Proteobacteria	Alphaproteobacteria	Rhizobiales	Rhizobiaceae	Pseudaminobacter	0.00	0.00	0.00	0.01	0.00	0.00	0.00	0.00	A	P	A	A
Bacteria	Proteobacteria	Alphaproteobacteria	Rhizobiales	Rhizobiaceae	Shinella	0.01	0.01	0.06	0.18	0.01	0.02	0.00	0.00	P	P	P	A
Bacteria	Proteobacteria	Alphaproteobacteria	Rhizobiales	Rhizobiaceae	Unknown	0.07	0.10	0.21	0.68	0.01	0.03	0.00	0.01	P	P	P	P
Bacteria	Proteobacteria	Alphaproteobacteria	Rhizobiales	Rhizobiales Incertae Sedis	Phreatobacter	0.00	0.00	0.01	0.03	0.00	0.02	0.00	0.00	A	P	P	A
Bacteria	Proteobacteria	Alphaproteobacteria	Rhizobiales	Xanthobacteraceae	Bradyrhizobium	0.00	0.00	0.03	0.07	0.00	0.01	0.01	0.04	A	P	P	P
Bacteria	Proteobacteria	Alphaproteobacteria	Rhizobiales	Xanthobacteraceae	Rhodopseudomonas	0.00	0.00	0.00	0.01	0.00	0.00	0.00	0.00	A	P	P	A
Bacteria	Proteobacteria	Alphaproteobacteria	Rhizobiales	Xanthobacteraceae	Unknown	0.00	0.00	0.00	0.01	0.00	0.00	0.00	0.00	A	P	A	A

Bacteria	Proteobacteria	Alphaproteobacteria	Rhodobacterales	Rhodobacteraceae	Amaricoccus	0.00	0.00	0.00	0.00	0.00	0.00	0.01	0.04	A	A	A	P
Bacteria	Proteobacteria	Alphaproteobacteria	Rhodobacterales	Rhodobacteraceae	Haematobacter	0.00	0.00	0.00	0.00	0.00	0.00	0.00	0.01	A	A	A	P
Bacteria	Proteobacteria	Alphaproteobacteria	Rhodobacterales	Rhodobacteraceae	Paracoccus	0.00	0.00	0.00	0.01	0.03	0.03	0.03	0.06	P	P	P	P
Bacteria	Proteobacteria	Alphaproteobacteria	Rickettsiales	AB1	Unknown	0.00	0.00	0.00	0.00	0.00	0.00	0.00	0.01	A	A	A	P
Bacteria	Proteobacteria	Alphaproteobacteria	Rickettsiales	Anaplasmataceae	Wolbachia	1.78	2.96	0.57	0.98	75.60	21.45	78.79	24.31	P	P	P	P
Bacteria	Proteobacteria	Alphaproteobacteria	Sphingomonadales	Sphingomonadaceae	Novosphingobium	0.01	0.01	0.01	0.01	0.00	0.00	0.01	0.03	P	P	A	P
Bacteria	Proteobacteria	Alphaproteobacteria	Sphingomonadales	Sphingomonadaceae	Sphingobium	0.00	0.00	0.00	0.00	0.03	0.10	0.00	0.01	A	A	P	P
Bacteria	Proteobacteria	Alphaproteobacteria	Sphingomonadales	Sphingomonadaceae	Sphingomonas	0.01	0.03	0.05	0.05	0.58	0.95	0.13	0.23	P	P	P	P
Bacteria	Proteobacteria	Alphaproteobacteria	Sphingomonadales	Sphingomonadaceae	Sphingopyxis	0.01	0.02	0.00	0.00	0.01	0.02	0.00	0.01	P	A	P	P
Bacteria	Proteobacteria	Alphaproteobacteria	Unknown	Unknown	Unknown	0.00	0.00	0.00	0.00	0.00	0.01	0.00	0.00	A	A	P	A
Bacteria	Proteobacteria	Gammaproteobacteria	Burkholderiales	Burkholderiaceae	Burkholderia-Caballeronia-Paraburkholderia	0.00	0.00	0.00	0.00	0.00	0.01	0.00	0.00	A	A	P	P
Bacteria	Proteobacteria	Gammaproteobacteria	Burkholderiales	Burkholderiaceae	Cupriavidus	0.00	0.00	0.00	0.00	0.00	0.01	0.00	0.00	A	A	P	A
Bacteria	Proteobacteria	Gammaproteobacteria	Burkholderiales	Burkholderiaceae	Lautropia	0.00	0.00	0.00	0.00	0.01	0.02	0.00	0.00	A	P	P	P
Bacteria	Proteobacteria	Gammaproteobacteria	Burkholderiales	Chromobacteriaceae	Gulbenkiania	0.00	0.00	0.00	0.00	0.00	0.00	0.01	0.02	A	A	A	P
Bacteria	Proteobacteria	Gammaproteobacteria	Burkholderiales	Comamonadaceae	Acidovorax	0.00	0.01	0.04	0.07	0.00	0.00	0.00	0.00	P	P	A	P
Bacteria	Proteobacteria	Gammaproteobacteria	Burkholderiales	Comamonadaceae	Aquabacterium	0.12	0.18	0.09	0.09	0.37	0.31	0.52	0.86	P	P	P	P
Bacteria	Proteobacteria	Gammaproteobacteria	Burkholderiales	Comamonadaceae	Caldimonas	0.01	0.03	0.00	0.00	0.00	0.01	0.00	0.00	P	A	P	A
Bacteria	Proteobacteria	Gammaproteobacteria	Burkholderiales	Comamonadaceae	Comamonas	0.00	0.00	0.00	0.00	0.00	0.00	0.00	0.01	A	A	A	P
Bacteria	Proteobacteria	Gammaproteobacteria	Burkholderiales	Comamonadaceae	Curvibacter	0.00	0.00	0.00	0.00	0.01	0.03	0.00	0.00	A	A	P	A
Bacteria	Proteobacteria	Gammaproteobacteria	Burkholderiales	Comamonadaceae	Delftia	0.00	0.01	0.12	0.28	0.07	0.23	0.08	0.19	P	P	P	P
Bacteria	Proteobacteria	Gammaproteobacteria	Burkholderiales	Comamonadaceae	Hydrogenophaga	0.01	0.05	0.00	0.00	0.00	0.00	0.01	0.02	P	A	A	P
Bacteria	Proteobacteria	Gammaproteobacteria	Burkholderiales	Comamonadaceae	Leptothrix	0.00	0.00	0.00	0.00	0.02	0.03	0.04	0.09	P	P	P	P
Bacteria	Proteobacteria	Gammaproteobacteria	Burkholderiales	Comamonadaceae	Mitsuaria	0.00	0.00	0.00	0.00	0.00	0.00	0.00	0.00	A	A	P	A
Bacteria	Proteobacteria	Gammaproteobacteria	Burkholderiales	Comamonadaceae	Ottowia	0.00	0.00	0.00	0.01	0.00	0.00	0.00	0.00	A	P	A	A
Bacteria	Proteobacteria	Gammaproteobacteria	Burkholderiales	Comamonadaceae	Paucibacter	0.00	0.00	0.00	0.01	0.00	0.00	0.00	0.00	A	P	A	A
Bacteria	Proteobacteria	Gammaproteobacteria	Burkholderiales	Comamonadaceae	Pelomonas	0.00	0.00	0.00	0.01	0.01	0.03	0.01	0.03	A	P	P	P

Bacteria	Proteobacteria	Gammaproteobacteria	Burkholderiales	Comamonadaceae	Polaromonas	0.00	0.00	0.00	0.00	0.00	0.00	0.00	0.01	A	A	A	P
Bacteria	Proteobacteria	Gammaproteobacteria	Burkholderiales	Comamonadaceae	Pseudacidovorax	0.00	0.01	0.00	0.00	0.18	0.26	0.02	0.05	P	A	P	P
Bacteria	Proteobacteria	Gammaproteobacteria	Burkholderiales	Comamonadaceae	Ramlibacter	0.00	0.00	0.00	0.00	0.00	0.00	0.00	0.00	A	A	P	A
Bacteria	Proteobacteria	Gammaproteobacteria	Burkholderiales	Comamonadaceae	Tepidimonas	0.00	0.00	0.03	0.06	0.05	0.14	0.28	0.61	A	P	P	P
Bacteria	Proteobacteria	Gammaproteobacteria	Burkholderiales	Comamonadaceae	Unknown	0.00	0.00	0.00	0.00	0.00	0.00	0.01	0.02	P	A	P	P
Bacteria	Proteobacteria	Gammaproteobacteria	Burkholderiales	Comamonadaceae	Variovorax	0.00	0.01	0.00	0.00	0.04	0.07	0.29	1.04	P	A	P	P
Bacteria	Proteobacteria	Gammaproteobacteria	Burkholderiales	Comamonadaceae	Xylophilus	0.00	0.00	0.00	0.01	0.00	0.00	0.00	0.00	A	P	A	A
Bacteria	Proteobacteria	Gammaproteobacteria	Burkholderiales	Hydrogenophilaceae	Unknown	0.00	0.00	0.00	0.00	0.00	0.01	0.00	0.00	A	A	P	A
Bacteria	Proteobacteria	Gammaproteobacteria	Burkholderiales	Neisseriaceae	Neisseria	0.00	0.00	0.00	0.01	0.02	0.05	0.00	0.00	A	P	P	P
Bacteria	Proteobacteria	Gammaproteobacteria	Burkholderiales	Neisseriaceae	Unknown	0.00	0.00	0.00	0.00	0.01	0.02	0.00	0.01	A	A	P	P
Bacteria	Proteobacteria	Gammaproteobacteria	Burkholderiales	Oxalobacteraceae	Duganella	0.00	0.00	0.00	0.00	0.00	0.00	0.00	0.01	A	A	A	P
Bacteria	Proteobacteria	Gammaproteobacteria	Burkholderiales	Oxalobacteraceae	Janthinobacterium	0.00	0.00	0.00	0.00	0.00	0.00	0.00	0.01	A	A	A	P
Bacteria	Proteobacteria	Gammaproteobacteria	Burkholderiales	Oxalobacteraceae	Massilia	0.01	0.01	0.02	0.03	0.06	0.06	0.08	0.12	P	P	P	P
Bacteria	Proteobacteria	Gammaproteobacteria	Burkholderiales	Oxalobacteraceae	Noviherbaspirillum	0.00	0.00	0.00	0.00	0.00	0.00	0.00	0.01	A	A	A	P
Bacteria	Proteobacteria	Gammaproteobacteria	Burkholderiales	Rhodocyclaceae	Azospira	0.00	0.01	0.00	0.00	0.00	0.00	0.01	0.03	P	A	P	P
Bacteria	Proteobacteria	Gammaproteobacteria	Burkholderiales	Rhodocyclaceae	Dechloromonas	0.01	0.04	0.00	0.00	0.01	0.03	0.00	0.00	P	A	P	P
Bacteria	Proteobacteria	Gammaproteobacteria	Burkholderiales	Rhodocyclaceae	Thauera	0.00	0.01	0.00	0.00	0.00	0.00	0.00	0.00	P	A	A	A
Bacteria	Proteobacteria	Gammaproteobacteria	Burkholderiales	Rhodocyclaceae	Unknown	0.00	0.00	0.00	0.00	0.00	0.01	0.00	0.00	A	A	P	A
Bacteria	Proteobacteria	Gammaproteobacteria	Burkholderiales	SC-I-84	Unknown	0.00	0.00	0.00	0.01	0.00	0.00	0.00	0.00	A	P	A	A
Bacteria	Proteobacteria	Gammaproteobacteria	Coxiellales	Coxiellaceae	Coxiella	0.00	0.00	0.00	0.00	0.00	0.00	0.01	0.04	A	P	A	P
Bacteria	Proteobacteria	Gammaproteobacteria	Enterobacterales	Aeromonadaceae	Aeromonas	0.00	0.00	0.00	0.00	0.00	0.00	0.00	0.00	A	A	A	P
Bacteria	Proteobacteria	Gammaproteobacteria	Enterobacterales	Enterobacteriaceae	Enterobacter	0.00	0.00	8.03	14.27	1.48	2.61	0.01	0.02	P	P	P	P
Bacteria	Proteobacteria	Gammaproteobacteria	Enterobacterales	Enterobacteriaceae	Escherichia-Shigella	0.15	0.09	0.26	0.22	1.76	1.62	0.95	0.98	P	P	P	P
Bacteria	Proteobacteria	Gammaproteobacteria	Enterobacterales	Enterobacteriaceae	Klebsiella	0.00	0.00	0.79	1.39	0.97	1.57	0.01	0.05	A	P	P	P
Bacteria	Proteobacteria	Gammaproteobacteria	Enterobacterales	Enterobacteriaceae	Salmonella	0.00	0.00	1.67	2.87	0.21	0.56	0.00	0.01	A	P	P	P
Bacteria	Proteobacteria	Gammaproteobacteria	Enterobacterales	Enterobacteriaceae	Unknown	0.00	0.00	5.26	9.13	0.65	1.73	0.01	0.02	A	P	P	P
Bacteria	Proteobacteria	Gammaproteobacteria	Enterobacterales	Erwiniaceae	Erwinia	0.00	0.00	0.00	0.00	0.00	0.00	0.00	0.01	A	A	A	P

Bacteria	Proteobacteria	Gammaproteobacteria	Enterobacterales	Erwiniaceae	Pantoea	0.00	0.00	0.00	0.00	0.02	0.06	0.00	0.00	A	A	P	A
Bacteria	Proteobacteria	Gammaproteobacteria	Enterobacterales	Erwiniaceae	Unknown	0.00	0.00	0.00	0.00	0.01	0.04	0.00	0.00	A	A	P	A
Bacteria	Proteobacteria	Gammaproteobacteria	Enterobacterales	Pasteurellaceae	Actinobacillus	0.00	0.00	0.00	0.00	0.03	0.07	0.00	0.00	A	A	P	A
Bacteria	Proteobacteria	Gammaproteobacteria	Enterobacterales	Pasteurellaceae	Aggregatibacter	0.00	0.00	0.00	0.00	0.00	0.00	0.00	0.01	A	A	A	P
Bacteria	Proteobacteria	Gammaproteobacteria	Enterobacterales	Pasteurellaceae	Haemophilus	0.00	0.01	0.00	0.00	0.03	0.09	0.00	0.00	P	A	P	A
Bacteria	Proteobacteria	Gammaproteobacteria	Enterobacterales	Pasteurellaceae	Unknown	0.00	0.00	0.00	0.00	0.00	0.00	0.00	0.00	A	A	P	A
Bacteria	Proteobacteria	Gammaproteobacteria	Enterobacterales	Shewanellaceae	Shewanella	0.00	0.00	0.00	0.00	0.00	0.00	0.00	0.00	A	A	A	P
Bacteria	Proteobacteria	Gammaproteobacteria	Enterobacterales	Unknown	Unknown	0.00	0.00	0.00	0.00	0.00	0.01	0.00	0.00	P	P	P	A
Bacteria	Proteobacteria	Gammaproteobacteria	Enterobacterales	Yersiniaceae	Serratia	0.00	0.00	0.00	0.02	0.00	0.01	0.01	0.03	A	P	P	P
Bacteria	Proteobacteria	Gammaproteobacteria	Legionellales	Legionellaceae	Legionella	0.00	0.00	0.00	0.00	0.00	0.00	0.01	0.02	A	A	A	P
Bacteria	Proteobacteria	Gammaproteobacteria	Pseudomonadales	Moraxellaceae	Acinetobacter	0.00	0.00	0.62	1.04	0.73	1.16	1.82	3.30	P	P	P	P
Bacteria	Proteobacteria	Gammaproteobacteria	Pseudomonadales	Moraxellaceae	Enhydrobacter	0.02	0.03	0.01	0.02	0.07	0.13	0.04	0.09	P	P	P	P
Bacteria	Proteobacteria	Gammaproteobacteria	Pseudomonadales	Moraxellaceae	Moraxella	0.00	0.00	0.00	0.00	0.00	0.00	0.00	0.01	A	A	A	P
Bacteria	Proteobacteria	Gammaproteobacteria	Pseudomonadales	Moraxellaceae	Perlucidibaca	0.00	0.01	0.00	0.00	0.00	0.00	0.00	0.00	P	A	A	A
Bacteria	Proteobacteria	Gammaproteobacteria	Pseudomonadales	Pseudomonadaceae	Pseudomonas	0.00	0.01	0.06	0.14	0.28	0.53	0.14	0.33	P	P	P	P
Bacteria	Proteobacteria	Gammaproteobacteria	Salinisphaerales	Solimonadaceae	Alkanibacter	0.00	0.00	0.00	0.00	0.00	0.00	0.01	0.03	A	A	A	P
Bacteria	Proteobacteria	Gammaproteobacteria	Salinisphaerales	Solimonadaceae	Hydrocarboniphaga	0.00	0.00	0.00	0.00	0.01	0.01	0.02	0.08	P	P	P	P
Bacteria	Proteobacteria	Gammaproteobacteria	Salinisphaerales	Solimonadaceae	Nevskia	0.00	0.00	0.00	0.00	0.00	0.01	0.00	0.00	A	A	P	A
Bacteria	Proteobacteria	Gammaproteobacteria	Unknown	Unknown	Unknown	0.00	0.00	0.00	0.00	0.00	0.01	0.00	0.00	A	A	P	A
Bacteria	Proteobacteria	Gammaproteobacteria	Xanthomonadales	Rhodanobacteraceae	Chiayiivirga	0.00	0.00	0.00	0.00	0.00	0.00	0.00	0.00	A	A	P	A
Bacteria	Proteobacteria	Gammaproteobacteria	Xanthomonadales	Rhodanobacteraceae	Dokdonella	0.00	0.00	0.00	0.00	0.00	0.00	0.00	0.00	A	P	A	A
Bacteria	Proteobacteria	Gammaproteobacteria	Xanthomonadales	Rhodanobacteraceae	Unknown	0.00	0.00	0.00	0.00	0.00	0.01	0.00	0.00	A	A	P	A
Bacteria	Proteobacteria	Gammaproteobacteria	Xanthomonadales	Xanthomonadaceae	Pseudoxanthomonas	0.00	0.00	0.00	0.00	0.01	0.02	0.05	0.09	P	A	P	P
Bacteria	Proteobacteria	Gammaproteobacteria	Xanthomonadales	Xanthomonadaceae	Stenotrophomonas	0.00	0.00	0.00	0.00	0.00	0.01	0.01	0.03	A	P	P	P
Bacteria	Proteobacteria	Gammaproteobacteria	Xanthomonadales	Xanthomonadaceae	Vulcaniibacterium	0.00	0.00	0.01	0.03	0.01	0.04	0.08	0.13	A	P	P	P
Bacteria	Proteobacteria	Gammaproteobacteria	Xanthomonadales	Xanthomonadaceae	Xanthomonas	0.00	0.00	0.00	0.00	0.00	0.01	0.00	0.00	A	A	P	A
Bacteria	Proteobacteria	Unknown	Unknown	Unknown	Unknown	0.00	0.00	0.00	0.00	0.02	0.04	0.03	0.07	A	A	P	P

Bacteria	Unknown	Unknown	Unknown	Unknown	Unknown	0.01	0.02	0.05	0.09	0.69	0.53	0.83	1.28	P	P	P	P
Bacteria	Verrucomicrobiota	Chlamydiae	Chlamydiales	Parachlamydiaceae	Candidatus Protochlamydia	0.00	0.00	0.00	0.00	0.00	0.00	0.00	0.00	A	P	A	A
Bacteria	Verrucomicrobiota	Chlamydiae	Chlamydiales	Parachlamydiaceae	Neochlamydia	0.00	0.00	0.00	0.00	0.00	0.00	0.00	0.00	A	P	A	A
Bacteria	Verrucomicrobiota	Verrucomicrobiae	Chthoniobacterales	Terrimicrobiaceae	Terrimicrobium	0.00	0.00	0.00	0.00	0.00	0.00	0.01	0.03	A	A	A	P
Bacteria	Verrucomicrobiota	Verrucomicrobiae	Opitutales	Opitutaceae	Lacunisphaera	0.00	0.00	0.00	0.00	0.00	0.00	0.00	0.00	A	A	A	P
Bacteria	Verrucomicrobiota	Verrucomicrobiae	Opitutales	Opitutaceae	Unknown	0.00	0.00	0.00	0.00	0.00	0.00	0.00	0.01	A	A	A	P
Bacteria	Verrucomicrobiota	Verrucomicrobiae	Verrucomicrobiales	Akkermansiaceae	Akkermansia	0.00	0.00	0.00	0.00	0.00	0.00	0.00	0.00	A	A	A	P
Bacteria	Verrucomicrobiota	Verrucomicrobiae	Verrucomicrobiales	Rubritaleaceae	Luteolibacter	0.00	0.00	0.00	0.00	0.00	0.00	0.02	0.07	A	A	A	P
Bacteria	WPS-2	Unknown	Unknown	Unknown	Unknown	0.00	0.00	0.00	0.00	0.00	0.00	0.00	0.02	A	A	A	P
Eukaryota	Unknown	Unknown	Unknown	Unknown	Unknown	0.00	0.00	0.00	0.00	0.03	0.08	0.00	0.00	A	A	P	P
Unknown	Unknown	Unknown	Unknown	Unknown	Unknown	0.00	0.00	0.01	0.01	0.04	0.05	0.04	0.06	A	P	P	P
