# Supplementary material for: Carry-over effects of Bacillus thuringiensis on tolerant Aedes albopictus mosquitoes
Source: Parasit Vectors. 2024 Nov 7;17:456. doi: 10.1186/s13071-024-06556-3 (PMC11545555; doi:10.1186/s13071-024-06556-3)
Supplement: Supplementary file 11 — Additional file 11: Table S7. Common bacterial genera between groups and their average relative abundance. [file 13071_2024_6556_MOESM11_ESM.pdf]

Additional File 8: Table S5. Top 50 most abundant genera based on relative abundance.

						LC		LB		AC		AB	
**Kingdom**	**Phylum**	**Class**	**Order**	**Family**	**Genus**	**Mean_Abundance_LC**	**SD_Abundance_LC**	**Mean_Abundance_LB**	**SD_Abundance_LB**	**Mean_Abundance_AC**	**SD_Abundance_AC**	**Mean_Abundance_AB**	**SD_Abundance_AB**
Bacteria	Proteobacteria	Alphaproteobacteria	Rickettsiales	Anaplasmataceae	Wolbachia	1.78	2.96	0.57	0.98	75.60	21.45	78.79	24.31
Bacteria	Actinobacteriota	Actinobacteria	Micrococcales	Microbacteriaceae	Microbacterium	82.57	6.75	48.27	27.47	0.05	0.09	0.07	0.17
Bacteria	Proteobacteria	Alphaproteobacteria	Rhizobiales	Kaistiaceae	Kaistia	8.33	4.76	18.65	17.66	0.00	0.01	0.00	0.00
Bacteria	Firmicutes	Bacilli	Bacillales	Bacillaceae	Geobacillus	0.42	0.68	4.04	7.21	9.98	15.12	10.92	16.12
Bacteria	Proteobacteria	Gammaproteobacteria	Enterobacterales	Enterobacteriaceae	Enterobacter	0.00	0.00	8.03	14.27	1.48	2.61	0.01	0.02
Bacteria	Firmicutes	Bacilli	Staphylococcales	Staphylococcaceae	Staphylococcus	0.10	0.09	0.18	0.16	0.75	0.94	0.86	1.11
Bacteria	Proteobacteria	Alphaproteobacteria	Acetobacterales	Acetobacteraceae	Tanticharoenia	0.00	0.00	0.00	0.00	1.57	3.59	0.35	0.63
Bacteria	Proteobacteria	Gammaproteobacteria	Enterobacterales	Enterobacteriaceae	Salmonella	0.00	0.00	1.67	2.87	0.21	0.56	0.00	0.01
Bacteria	Proteobacteria	Gammaproteobacteria	Enterobacterales	Enterobacteriaceae	Klebsiella	0.00	0.00	0.79	1.39	0.97	1.57	0.01	0.05
Bacteria	Unknown	Unknown	Unknown	Unknown	Unknown	0.01	0.02	0.05	0.09	0.69	0.53	0.83	1.28
Bacteria	Proteobacteria	Alphaproteobacteria	Acetobacterales	Acetobacteraceae	Rubritepida	4.72	3.83	0.97	0.85	0.00	0.01	0.00	0.00
Bacteria	Proteobacteria	Gammaproteobacteria	Enterobacterales	Enterobacteriaceae	Unknown	0.00	0.00	5.26	9.13	0.65	1.73	0.01	0.02
Bacteria	Bacteroidota	Bacteroidia	Sphingobacteriales	Sphingobacteriaceae	Pedobacter	0.00	0.01	0.00	0.01	0.40	1.48	0.95	3.39
Bacteria	Bacteroidota	Bacteroidia	Flavobacteriales	Weeksellaceae	Chryseobacterium	0.02	0.03	0.39	0.92	0.52	1.87	0.42	1.16
Bacteria	Proteobacteria	Alphaproteobacteria	Rhizobiales	Rhizobiaceae	Allorhizobium-Neorhizobium-Pararhizobium-Rhizobium	0.05	0.07	0.53	1.52	0.70	1.62	0.02	0.04
Bacteria	Firmicutes	Bacilli	Bacillales	Bacillaceae	Bacillus	0.00	0.00	4.65	5.69	0.02	0.04	0.02	0.03
Bacteria	Proteobacteria	Gammaproteobacteria	Burkholderiales	Comamonadaceae	Aquabacterium	0.12	0.18	0.09	0.09	0.37	0.31	0.52	0.86
Bacteria	Firmicutes	Bacilli	Brevibacillales	Brevibacillaceae	Brevibacillus	0.07	0.16	0.16	0.28	0.69	1.02	0.20	0.40
Bacteria	Proteobacteria	Alphaproteobacteria	Rhizobiales	Beijerinckiaceae	Methylobacterium-Methylorubrum	0.13	0.21	0.58	0.71	0.15	0.22	0.16	0.27
Bacteria	Proteobacteria	Gammaproteobacteria	Pseudomonadales	Moraxellaceae	Acinetobacter	0.00	0.00	0.62	1.04	0.73	1.16	1.82	3.30
Bacteria	Proteobacteria	Gammaproteobacteria	Enterobacterales	Enterobacteriaceae	Escherichia-Shigella	0.15	0.09	0.26	0.22	1.76	1.62	0.95	0.98
Bacteria	Proteobacteria	Alphaproteobacteria	Sphingomonadales	Sphingomonadaceae	Sphingomonas	0.01	0.03	0.04	0.05	0.58	0.95	0.13	0.23
Bacteria	Actinobacteriota	Actinobacteria	Micrococcales	Beutenbergiaceae	Miniimonas	1.19	1.19	1.82	3.38	0.00	0.00	0.00	0.00

Bacteria	Bdellovibrionota	Bdellovibrionia	Bacteriovoracales	Bacteriovoracaceae	Peredibacter	0.00	0.00	0.62	1.40	0.00	0.01	0.00	0.00
Bacteria	Proteobacteria	Alphaproteobacteria	Rhizobiales	Beijerinckiaceae	Bosea	0.01	0.02	0.46	0.97	0.04	0.10	0.00	0.01
Bacteria	Proteobacteria	Gammaproteobacteria	Pseudomonadales	Pseudomonadaceae	Pseudomonas	0.00	0.01	0.06	0.14	0.28	0.53	0.14	0.33
Bacteria	Actinobacteriota	Actinobacteria	Propionibacteriales	Nocardioidaceae	Nocardioides	0.00	0.00	0.01	0.03	0.00	0.01	0.42	1.42
Bacteria	Proteobacteria	Gammaproteobacteria	Burkholderiales	Comamonadaceae	Tepidimonas	0.00	0.00	0.03	0.06	0.05	0.14	0.28	0.61
Bacteria	Proteobacteria	Gammaproteobacteria	Burkholderiales	Comamonadaceae	Variovorax	0.00	0.01	0.00	0.00	0.04	0.07	0.29	1.04
Bacteria	Proteobacteria	Gammaproteobacteria	Burkholderiales	Comamonadaceae	Delftia	0.00	0.01	0.12	0.28	0.07	0.23	0.08	0.19
Bacteria	Proteobacteria	Alphaproteobacteria	Rhizobiales	Rhizobiaceae	Unknown	0.07	0.10	0.21	0.68	0.01	0.03	0.00	0.01
Bacteria	Proteobacteria	Alphaproteobacteria	Rhizobiales	Rhizobiaceae	Aminobacter	0.00	0.00	0.28	0.90	0.00	0.00	0.00	0.00
Bacteria	Actinobacteriota	Actinobacteria	Propionibacteriales	Propionibacteriaceae	Cutibacterium	0.01	0.01	0.02	0.03	0.08	0.09	0.11	0.14
Bacteria	Proteobacteria	Gammaproteobacteria	Burkholderiales	Comamonadaceae	Pseudacidovorax	0.00	0.01	0.00	0.00	0.18	0.26	0.02	0.05
Bacteria	Proteobacteria	Gammaproteobacteria	Burkholderiales	Oxalobacteraceae	Massilia	0.01	0.01	0.02	0.03	0.06	0.06	0.08	0.12
Bacteria	Actinobacteriota	Actinobacteria	Corynebacteriales	Corynebacteriaceae	Corynebacterium	0.00	0.01	0.01	0.02	0.04	0.05	0.11	0.17
Bacteria	Firmicutes	Bacilli	Lactobacillales	Streptococcaceae	Streptococcus	0.00	0.00	0.01	0.03	0.06	0.11	0.09	0.16
Bacteria	Actinobacteriota	Actinobacteria	Corynebacteriales	Nocardiaceae	Rhodococcus	0.01	0.02	0.00	0.01	0.05	0.08	0.09	0.14
Bacteria	Actinobacteriota	Actinobacteria	Corynebacteriales	Corynebacteriaceae	Lawsonella	0.00	0.01	0.02	0.04	0.03	0.04	0.09	0.19
Bacteria	Proteobacteria	Alphaproteobacteria	Rhizobiales	Rhizobiaceae	Mesorhizobium	0.00	0.00	0.09	0.27	0.05	0.11	0.00	0.01
Bacteria	Proteobacteria	Gammaproteobacteria	Pseudomonadales	Moraxellaceae	Enhydrobacter	0.02	0.03	0.01	0.02	0.07	0.13	0.04	0.09
Bacteria	Proteobacteria	Gammaproteobacteria	Xanthomonadales	Xanthomonadaceae	Vulcaniibacterium	0.00	0.00	0.01	0.03	0.01	0.04	0.08	0.13
Bacteria	Firmicutes	Bacilli	Lactobacillales	Lactobacillaceae	Lactobacillus	0.02	0.02	0.00	0.02	0.04	0.05	0.04	0.07
Bacteria	Bacteroidota	Bacteroidia	Cytophagales	Cytophagaceae	Siphonobacter	0.00	0.00	0.06	0.10	0.05	0.13	0.00	0.01
Bacteria	Bacteroidota	Bacteroidia	Sphingobacteriales	Sphingobacteriaceae	Sphingobacterium	0.00	0.00	0.00	0.00	0.00	0.01	0.09	0.33
Unknown	Unknown	Unknown	Unknown	Unknown	Unknown	0.00	0.00	0.01	0.01	0.04	0.05	0.04	0.06
Bacteria	Actinobacteriota	Actinobacteria	Micrococcales	Microbacteriaceae	Galbitalea	0.04	0.06	0.03	0.05	0.00	0.00	0.00	0.00
Bacteria	Proteobacteria	Alphaproteobacteria	Rhizobiales	Rhizobiaceae	Shinella	0.01	0.01	0.06	0.18	0.01	0.02	0.00	0.00
Bacteria	Proteobacteria	Gammaproteobacteria	Burkholderiales	Comamonadaceae	Leptothrix	0.00	0.00	0.00	0.00	0.02	0.03	0.04	0.09
Bacteria	Proteobacteria	Alphaproteobacteria	Rhodobacterales	Rhodobacteraceae	Paracoccus	0.00	0.00	0.00	0.01	0.03	0.03	0.03	0.06
