# Supplementary material for: Carry-over effects of Bacillus thuringiensis on tolerant Aedes albopictus mosquitoes
Source: Parasit Vectors. 2024 Nov 7;17:456. doi: 10.1186/s13071-024-06556-3 (PMC11545555; doi:10.1186/s13071-024-06556-3)
Supplement: Supplementary file 12 — Additional file 12: Table S8. Statistical analysis of differentially abundant genus in each experimental group. [file 13071_2024_6556_MOESM12_ESM.pdf]

**Additional File 9: Table S6.** Comparison of diversity metrics of the gut microbiota in LB, LC, AB, and AC samples.

**A) Kruskal-Wallis pairwise comparison of alpha diversity indices**

| | | LC | AC | LB |
|---|---|---|---|---|
| Control | LC | | | |
| | AC | 21.67 **(p-value<0.001)** | | |
| Bti-tolerant | LB | 16.90 **(p-value<0.001)** | 13.44 **(p-value<0.001)** | |
| | AB | 21.67 **(p-value<0.001)** | 2.89 **(p-value=0.089)** | 7.32 **(p-value<0.005)** |

**B) Pairwise PerMANOVA analysis of Bray-Curtis distances based on Pseudo-F test statistic**

| | | LC | AC | LB |
|---|---|---|---|---|
| Control | LC | | | |
| | AC | 45.31 **(p-value<0.001)** | | |
| Bti-tolerant | LB | 8.38 **(p-value<0.001)** | 16.67 **(p-value<0.001)** | |
| | AB | 50.64 **(p-value<0.001)** | 1.72 **(p-value<0.043)** | 19.89 **(p-value<0.001)** |
